# Molecular Identification of Commercialized Medicinal Plants in Southern Morocco

**DOI:** 10.1371/journal.pone.0039459

**Published:** 2012-06-27

**Authors:** Anneleen Kool, Hugo J. de Boer, Åsa Krüger, Anders Rydberg, Abdelaziz Abbad, Lars Björk, Gary Martin

**Affiliations:** 1 Department of Systematic Biology, Evolutionary Biology Centre, Uppsala University, Uppsala, Sweden; 2 Department of Botany, Stockholm University, Stockholm, Sweden; 3 Laboratory of Biotechnology, Protection and Valorisation of Plant Resources, Faculty of Science Semlalia, Cadi Ayyad University, Marrakech, Morocco; 4 Global Diversity Foundation, Dar Ylane, Marrakech, Morocco; American Museum of Natural History, United States of America

## Abstract

**Background:**

Medicinal plant trade is important for local livelihoods. However, many medicinal plants are difficult to identify when they are sold as roots, powders or bark. DNA barcoding involves using a short, agreed-upon region of a genome as a unique identifier for species– ideally, as a global standard.

**Research Question:**

What is the functionality, efficacy and accuracy of the use of barcoding for identifying root material, using medicinal plant roots sold by herbalists in Marrakech, Morocco, as a test dataset.

**Methodology:**

In total, 111 root samples were sequenced for four proposed barcode regions *rpoC1*, *psbA-trnH*, *matK* and ITS. Sequences were searched against a tailored reference database of Moroccan medicinal plants and their closest relatives using BLAST and Blastclust, and through inference of RAxML phylograms of the aligned market and reference samples.

**Principal Findings:**

Sequencing success was high for *rpoC1*, *psbA-trnH*, and ITS, but low for *matK*. Searches using *rpoC1* alone resulted in a number of ambiguous identifications, indicating insufficient DNA variation for accurate species-level identification. Combining *rpoC1*, *psbA-trnH* and ITS allowed the majority of the market samples to be identified to genus level. For a minority of the market samples, the barcoding identification differed significantly from previous hypotheses based on the vernacular names.

**Conclusions/Significance:**

Endemic plant species are commercialized in Marrakech. Adulteration is common and this may indicate that the products are becoming locally endangered. Nevertheless the majority of the traded roots belong to species that are common and not known to be endangered. A significant conclusion from our results is that unknown samples are more difficult to identify than earlier suggested, especially if the reference sequences were obtained from different populations. A global barcoding database should therefore contain sequences from different populations of the same species to assure the reference sequences characterize the species throughout its distributional range.

## Introduction

### 1.1 Marrakech Medicinal Plant Trade and the Moroccan Herbal Pharmacopoeia

Traditional medicine has played an important role in many North African societies, and continues to do so today [1]. This is evident not least in the Moroccan city of Marrakech, situated at a crossroads of trade routes between the High Atlas Mountains and surrounding coastal plains.

**Figure 1:**
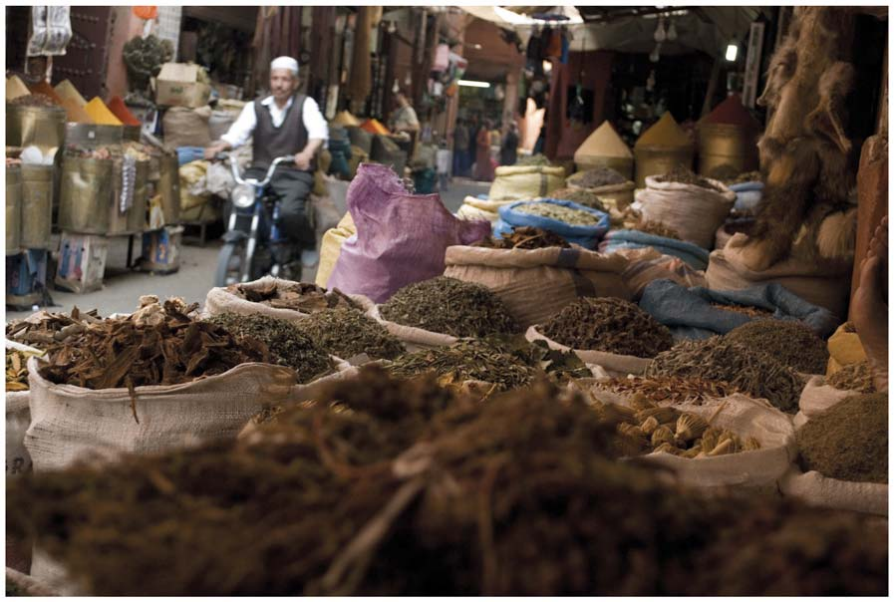
Typical herbalist shop in the medina of Marrakech.

The traditional equivalent of the doctor in Moroccan medicine is the herbalist – a profession that continues to be practiced in Marrakech, manifested by the herbalist-owned drug stores that line the market districts of the *medina*, or old town (Fig. 1). In these shops, Marrakech herbalists stock a variety of plant parts and plant-derived products, sold either separately or in mixtures. In general, these plant parts are harvested in the wild [2], by specialized collectors and reach the herbalists through middlemen and wholesalers [3].

An important part of the plant inventory of Moroccan herbalists consists of barks and roots, which typically possess few physical characteristics that enable accurate morphology-based identification. All herbalists are able to provide information about the local name of a plant product, its medicinal uses and origins, but this information may be imprecise, or insufficient for species identification purposes, especially considering that herbalists often do not possess knowledge of medicinal plants in the wild [3]. Some medicinal products have multiple synonymous names, and in other cases the same vernacular name is applied to multiple plant species [4]. In other words, confirming the identity of a root sample bought from these herbalists has so far presented a challenge. In addition, since the collection of roots usually requires the whole plant to be dug up, the trade of medicinal roots has a large impact on natural plant populations [5], [6].

The identity of the plants being sold in these markets has conservational as well as medical implications. For example, rare or endangered species could inadvertently be collected if they are easily confused with their more abundant relatives. Likewise, increasing demands for medicinal products may lead to local over-harvesting extinction of otherwise non-threatened plant species. Misidentified collections could also lead to the introduction of toxic or otherwise unsuitable species to the market, with potential health risks to end-users [7], [8]. For example Chinese star anise (*Illicium verum* Hook f.) is commonly used in herbal teas, whereas Japanese star anise (*I. anisatum* L.) causes neurotoxic effects in infants when used as a substitute for Chinese star anise [8]. In all cases, appropriate measures could be taken if a reliable method for species identification of medicinal plant products existed.

### 1.2 Molecular Identification

Species identification on the basis of DNA sequences has been done for some time, e.g. fungi [9], animals [10]–[13], plants [14]. Hebert et al. [15] proposed to use the mitochondrial gene *CO1* as the standard *barcode* for all animals, and this was readily adopted by the scientific community. Assessments have since shown that *CO1* can be used to distinguish over 90% of species in most animal groups [16], [17]. In recent years barcoding research has grown substantially, and worldwide efforts coordinated by the Consortium for the Barcode of Life (CBOL) are now being focused on retrieving barcode sequences from all organisms [18].

Barcoding in other major groups, such as plants, has developed at a markedly slower pace. Early on, it became clear that the mitochondrial genome evolves far too slowly in most plants to allow it to distinguish between species [19], [20]. Various genes and non-coding regions in the plastid genome have been put forward as alternatives [18], [19], [21]–[24]. In addition to being sufficiently fast evolving, a molecular barcode must also be flanked by conserved regions that can function as universal primer binding sites for PCR reactions [21]. A single barcoding locus combining these two traits has not been found for plants, and it appears that a combination of two or more, probably plastid, loci will almost certainly be required to approach the level of species discrimination and universality that *CO1* provides for animals [22]. In 2009, CBOL proposed *matK* and *rbcL* combined as a universal barcode for land plants, but with the option to supplement it with one or two other markers [18], for example *psbA-trnH* or ITS [25].

Most species concepts agree on species being evolving metapopulation lineages, but delimiting species is often more problematic [26]. The importance role of hybridization in plant speciation makes species delimitation in plants much more complicated than in animals [27]. Species delimitation based on molecular data in the light of coalescent theory is being developed but requires many accessions as well as many loci [28]. In an ideal situation, studies at population genetic level would have to be done for all species in a DNA barcoding database; this is far from being achieved at present and instead a more or less arbitrary cut-off value for sequence divergence is often used [29]–[31]. The main methodological problem with DNA barcoding remains that it is often impossible to tell the difference between interspecific sequence variation and intraspecific sequence variation [24], [32], [33]. But notably, difficulties in distinguishing between intra- and interspecific variation are a widespread problem in morphological species delimitation as well.

Even in animals molecular barcoding is problematic, since approximately 88% of the estimated 7.8 million animal species lack a formal description [34], [35], and adopting an arbitrary cut-off value for pairwise sequence divergence distance to speed up cataloguing these undescribed species would be disastrous for existing taxonomic treatments in animals [36]. Also in fungi, another group in which the vast majority of the taxa is undescribed, an arbitrary sequence divergence threshold for the nuclear ribosomal ITS region proved to be not feasible [37], [38]. The fields of molecular identification, DNA barcoding, and DNA taxonomy are still very much in development, and are certainly not without practical or theoretical problems.

Despite these problems, DNA barcoding has been applied to a broad range of problems, including taxonomic studies of cryptic taxa or species complexes, e.g. skipper butterflies [39]. Barcoding has also been used in ecological studies to survey animal diets through the analysis of plant remains in faeces [40], in identifying plant species from wood samples [41], and as a tool to control the cross-border trade of aquarium fish [42]. In addition molecular identification has been used in several studies on traditional medicine [7], [24], [43]–[46]. Barcoding lends itself particularly well to these forensic applications where only a small tissue sample from the organism is available for identification, or where the sample is degraded or has been processed.

Methods for matching an unknown query sequence with a reference database tend to be either based on sequence similarity like BLAST [47] (e.g. [48]) and Blastclust [49] (e.g. [50]), or on tree-based criteria [15], [36], [50], [51]. Several other alignment-free methods, e.g. DNA-BAR/DEGENBAR and ATIM, have been proposed, but these are reported to perform equally well as BLAST [48], [52]. Sequence similarity methods require a decision on a threshold at which a sequence is considered to belong to a certain taxon, which can be somewhat subjective and may be applicable to certain taxa but not to others [35], [36]. Tree-based methods, in which a query sequence is considered to belong to a certain taxon if it is found in a clade consisting of reference sequences for that taxon, have as a clear advantage that no cut-off value is necessary, but they do require an alignment of the query and reference sequences combined, which can be problematic for highly variable sequences [19]. Nonetheless, the success of any method used to assign sequences to a certain taxon is ultimately dependent on the taxonomic coverage of the reference database.

There is a wide variety of studies that assess the efficacy of molecular identification techniques by analysing the sequence variation within a large number of known samples [21], [32], [53], [54], or by identifying query sequences from the same dataset as the reference sequences [22], [50], [55], [56]. Studies using a separate query dataset to investigate the identification success of a certain marker or marker-combination is not commonly done. Gonzalez et al. [57] used a reference database created for a lowland rainforest area in French Guiana to identify saplings from the same area and reported a significantly lower identification success rate (70%) than most other studies due to low sequence variation in a few species-rich clades. A study on ingredients of commercial teas showed that *rbcL* and *matK* could identify roughly 70% of the ingredients in tea, but that sequence variation between closely related tea ingredients was in the same order of magnitude as sequencing error [58].

In this study we investigate which medicinal roots are commercialized in the souks of Marrakech using a regional reference database approach and sequence data from the plastid genome (*matK*, *psbA-trnH*, and *rpoC1*) as well as the nuclear genome (ITS). *RbcL*, albeit one of the standard plant DNA barcodes, was not included as its sequence variation is comparable to that of *rpoC1* [18], [22]. We compare using BLAST combined with additional data on the occurrence of the plant in Morocco, with the use of Blastclust and a RAxML analysis of the aligned query and reference sequences and were able to identify roughly half of the samples to species level and an additional third of the samples to genus level.

## Results

### 2.1 DNA Extraction, PCR and Sequencing Success

The standard extraction protocol worked for approximately 75% of the market and all but one of the reference samples. However, for 28 out of 111 market samples the extraction method consistently failed to yield PCR products.

Amplification of *matK* yielded PCR products for less than 30% of the reference specimens and *matK* was subsequently excluded as a potential barcode in this study, as was also done by Piredda et al. [50] and Sass et al. [48]. Sequencing success rates for the other three loci (*rpoC1*, *psbA-trnH*, and ITS) for both reference- and market samples are detailed in Table 1, and most roots were successfully sequenced for at least two of the regions (Data S1). *RpoC1* sequence lengths ranged from 409 to 545 bp, *psbA-trnH* sequence lengths from 141 to 658 bp, and ITS sequence lengths from 194 to 748 bp. The reference samples (Data S2), which were extracted from herbarium vouchers collected mainly in Morocco (Data S3), were consistently easier to sequence than the market samples.

**Table 1:** Sequencing success (%) per group.

	#^1^	*rpoC1*	*psbA-trnH*	ITS
**Reference samples**	**130**	**90.8%**	**80.0%**	**76.2%**
Monocots	18	66.7%	55.6%	50.0%
Eudicots	106	95.3%	85.0%	85.8%
Apiaceae	25	100.0%	88.0%	96.0%
Asteraceae	28	96.4%	82.1%	82.1%
Caryophyllaceae	7	100.0%	85.7%	71.4%
**Market samples**	**83**	**88.0%**	**74.7%**	**69.9%**
Monocots	13	69.0%	46.0%	15.0%
Eudicots	69	89.9%	81.2%	65.2%
Apiaceae	18	100.0%	77.7%	55.6%
Asteraceae	22	81.8%	86.3%	72.7%
Caryophyllaceae	8	87.5%	100.0%	75.0%

1 Including only samples from successful total DNA extraction.

A total of nine ITS sequences obtained from the market samples and ten of the reference ITS sequences turned out to have fungal contamination. Twenty-nine ITS sequences of the market samples and fourteen of the reference samples could not be used because of polymorphisms.

The extended reference databases, obtained through downloading all sequences that yielded an E-value of 0.0 in the initial BLAST searches consisted of 1864 (*rpoC1*), 2332 (*psbA-trnH*), and 3168 (ITS) sequences. The aligned rpoC1 dataset consisted of 652 aligned positions and the aligned datasets of *psbA-trnH* and ITS of 706, respectively 1327 aligned positions. All three alignments contained insertion-deletions (indels), but the aligned matrix of the coding region (*rpoC1*) contained significantly less indels than the ITS and *psbA-trnH* matrices. The RAxML phylograms (Data S4, S5, S6) and Blastclust output (Data S7, S8, S9) for all three datasets are presented in the Dataemental data.

**Figure 2:**
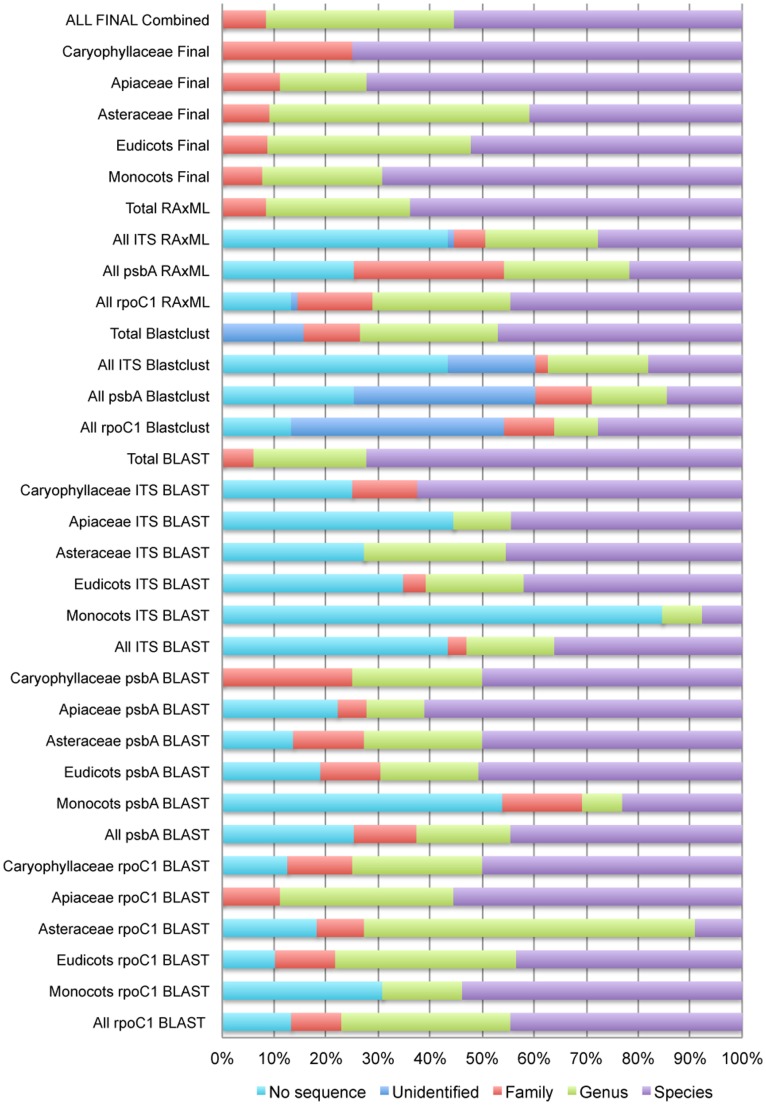
Relative identification success per marker, analysis method and taxonomic group.

The identification success was dependent on marker, identification method as well as taxonomic group (Fig. 2, Data S3). Blastclust analysis of the *psbA-trnH* data yielded fewest identifications (24 of 83 sequences identified to either species or genus level) whereas BLAST analysis of the *rpoC1* data was most successful (64 of 83 sequences identified to either species of genus level). The identification success was somewhat higher for monocots than for eudicots using *rpoC1* or ITS, whereas eudicots were more readily identified using *psbA-trnH*.

The identification of the market samples and how these identifications differ from those based on the pharmacopeia is presented in Table 2 and discussed in Data S10. In total 15 (18%) of the samples were identified as belonging to a different species than the one mentioned in the pharmacopoeia. Of these, ten belonged to a different genus than earlier hypothesized and five to a different family.

**Table 2:** Barcoding identifications and GenBank accession numbers in order of transcribed Arab product name.

Coll. No.^a^	Vernacular name^b^	Putative scientific name^c^	ID Confirmed	FINAL ID
EM449	‘Aqirqarha [good]	*Anacyclus pyrethrum*	Genus confirmed	*Anacyclus* sp.
EM408	‘Aqirqarha [highest]	*Anacyclus pyrethrum*	Genus confirmed	*Anacyclus* sp.
EM444	‘Aqirqarha [highest]	*Anacyclus pyrethrum*	Genus confirmed	*Anacyclus* sp.
EM448	‘Aqirqarha [worst]	*Anacyclus pyrethrum*	Genus confirmed	*Anacyclus* sp.
EM362	‘Aqirqarha [secondary]	*Anacyclus pyrethrum*	Different genus	*Catananche* sp.
EM361	‘Aqirqarha [highest]	*Anacyclus pyrethrum*	Different genus	*Catananche caespitosa*
EM416	‘Aqirqarha [secondary]	*Anacyclus pyrethrum*	Different genus	*Catananche* sp.
EM450	‘Aqirqarha [secondary]	*Anacyclus pyrethrum*	Genus confirmed	*Anacyclus* sp.
EM399	‘Arq assus	*Glycyrrhiza glabra*	Genus confirmed	*Glycyrrhiza* sp.
EM409	‘Arq assus lbldi	*Glycyrrhiza glabra*	Genus confirmed	*Glycyrrhiza* sp.
EM378	‘Arq assus lhchich	*Glycyrrhiza glabra*	Genus confirmed	*Glycyrrhiza* sp.
EM373	‘Arq assus lqash	*Glycyrrhiza glabra*	Genus confirmed	*Glycyrrhiza* sp.
EM357	‘Erq wadmi lbldi	*Armeria sp.*	Species confirmed	*Armeria* sp.
EM358	‘Erq wadmi rroumi	*Armeria sp.*	Species confirmed	*Armeria* sp.
EM429	‘Ud-mserser [highest]	*Polygonum aviculare Daucus crinitus*	Species confirmed	*Daucus crinitus*
EM453	‘Ud-mserser [highest]	*Polygonum aviculare Daucus crinitus*	Species confirmed	*Daucus crinitus*
EM417	‘Ud-mserser [secondary]	*Polygonum aviculare Daucus crinitus*	Family confirmed	*Thapsia* sp.
EM451	‘Ud-mserser [secondary]	*Polygonum aviculare Daucus crinitus*	Different genus	*Thapsia* sp.
EM437	Addad	*Carlina gummifera*	Genus confirmed	*Carlina gummifera*
EM374	Addad bjlftou	*Carlina gummifera*	*Species confirmed*	*Carlina gummifera*
EM397	Addad dkr	*Carlina gummifera*	Species confirmed	*Carlina gummifera*
EM380	Addad lmjllaf	*Carlina gummifera*	Species confirmed	*Carlina gummifera*
EM396	Addad ntwa	*Carlina gummifera*	Family confirmed	Asteraceae
EM431	‘Ansal	*Drimia maritima*	Species confirmed	*Drimia* sp.
EM446	As-susan	*Iris x germanica*	Genus confirmed	*Iris* sp.
EM365	Besbas lbldi	*Foeniculum vulgare*	Species confirmed	*Anethum foeniculoides Foeniculum vulgare*
EM387	Besbas lbldi	*Foeniculum vulgare*	Species confirmed	*Anethum foeniculoides Foeniculum vulgare*
EM369	Besbas lbldi	*Foeniculum vulgare*	Different family	*Echinops* sp.
EM366	Besbas lboustani	*Foeniculum vulgare*	Species confirmed	*Anethum foeniculoides Foeniculum vulgare*
EM372	Besbas lboustani	*Foeniculum vulgare*	Species confirmed	*Anethum foeniculoides Foeniculum vulgare*
EM404	Bid al-gul	*Mandragora autumnalis*	Different species	*Mandragora officinarum*
EM436	Buglam sahrawi	*Spergularia marginata*	Family confirmed	Caryophyllaceae
EM377	Bougoudz	Unidentified	Previously unknown	*Dioscorea communis*
EM452	Bougoudz	Unidentified	Previously unknown	*Dioscorea communis*
OA1	Bougoudz	Unidentified	Previously unknown	*Dioscorea communis*
OA2	Bougoudz	Unidentified	Previously unknown	*Dioscorea communis*
OA4	Bougoudz	Unidentified	Previously unknown	*Dioscorea communis*
EM447	Bu-zfur	*Daucus crinitus*	Different genus	*Kundmannia sicula*
EM405	Brztm	*Aristolochia fontanesii*	Genus confirmed	*Aristolochia* sp.
EM410	Bukbuka	*Colchicum autumnale*	Different family	*Bunium* sp.
EM434	Dbag lbldi	*Quercus sp.*	Species confirmed	*Quercus ilex*
EM414	Deryas	*Thapsia garganica*	Family confirmed	Apiaceae
EM371	Frifra	*Magydaris panacifolia*	Different genus	*Kundmannia sicula*
EM412	Frifra	*Magydaris panacifolia*	Different genus	*Anethum foeniculoides Foeniculum vulgare*
EM438	Fuwwa	*Rubia peregrina R. tinctorum*	Genus confirmed	*Rubia* sp.
EM379	Fuwwa lfrouguiyya	*Rubia peregrina R. tinctorum*	Family confirmed	Rubiaceae
EM390	Fuwwa lfrouguiyya	*Rubia peregrina R. tinctorum*	Genus confirmed	*Rubia* sp.
EM391	Fuwwa rqiqa (jbal nawahi mrrakch)	*Rubia peregrina R. tinctorum*	Different genus	*Galium* sp.
EM398	Fwila	*Erophaca baetica subsp. baetica*	Species confirmed	*Erophaca baetica* subsp. *baetica*
EM430	Horsef	*Cynara cardunculus*	Genus confirmed	*Cynara* sp.
EM395	Horsef rroumi	*Cynara cardunculus*	Genus confirmed	*Echinops spinosissimus*
EM402	L-fijel	*Ruta montana*	Species confirmed	*Ruta montana*
EM439	L-gseb	*Arundo donax*	Species confirmed	*Arundo donax*
EM443	L-gseb	*Arundo donax*	Species confirmed	*Arundo donax*
EM442	L-harmel	*Peganum harmala*	Different family	*Carlina brachylepis*
OA3	L-harmel	*Peganum harmala*	Different family	*Vitis* sp.
EM432	Lghzghaz	*Carlina involucrata*	Species confirmed	*Carlina brachylepis*
EM433	Lklkh	*Ferula communis*	Species confirmed	*Ferula communis*
EM435	Luwwaya	*Smilax aspera*	Species confirmed	*Smilax aspera*
EM382	Mgizla	*Eryngium triquetrum*	Genus confirmed	*Eryngium* sp.
EM424	Mgizla	*Eryngium triquetrum*	Genus confirmed	*Eryngium* sp.
EM422	Ndkhir	Unidentified	Previously unknown	*Dioscorea communis*
EM388	Nnjem lbori	*Cynodon dactylon*	Family confirmed	Poaceae
EM389	Nnjem lmawi	*Cynodon dactylon*	Different genus	*Panicum* sp.
EM427	Oudn lhllouf	*Pulicaria arabica*	Different species	*Pulicaria odora*
EM403	Sargina	*Corrigiola telephiifolia*	Species confirmed	*Corrigiola litoralis subsp. litoralis*
EM368	Sargina l3adia	*Corrigiola telephiifolia*	Different genus	*Silene mentagensis*
EM376	Sargina l3adia	*Corrigiola telephiifolia*	Species confirmed	*Corrigiola litoralis subsp. telephiifolia*
EM367	Sargina lmsouwsa	*Corrigiola telephiifolia*	Species confirmed	*Corrigiola litoralis subsp. telephiifolia*
EM421	Sargina lmsouwsa	*Corrigiola telephiifolia*	Species confirmed	*Corrigiola litoralis subsp. telephiifolia*
EM423	Sargina rrahmania	*Corrigiola telephiifolia*	Family confirmed	Caryophyllaceae
EM440	Ssder	*Ziziphus lotus*	Species confirmed	*Ziziphus lotus*
EM413	Tafga	*Rhaponticum acaule*	Genus confirmed	*Rhaponticum sp.*
OA10	Tafga	*Rhaponticum acaule*	Family confirmed	Asteraceae
EM411	Talh	*Acacia* sp.	Species confirmed	*Acacia gummifera*
EM363	Talh dkr	*Acacia* sp.	Species confirmed	*Acacia gummifera*
EM364	Talh ntwa	*Acacia* sp.	Species confirmed	*Acacia gummifera*
EM407	Taskra	*Echinops spinosissimus*	Genus confirmed	*Echinops* sp.
EM356	Terta	*Withania frutescens*	Different family	*Kundmannia sicula*
OA11	Terta	*Withania frutescens*	Genus confirmed	*Withania* sp.
OA8	Terta	*Withania frutescens*	Genus confirmed	*Withania* sp.
EM428	Tigigest	*Silene sp.*	Species confirmed	*Silene vulgaris*
EM425	Zziyata	*Kundmannia sicula*, *Limoniastrum guyonianum*, *L. ifniense*, *Conium maculatum, Apium nodiflorum, Polygonum maritimum*	Species confirmed	*Kundmannia sicula*

## Discussion

### 3.1 Analyses and Role of Markers, Methods, and Taxonomic Group

#### 3.1.1 RpoC1

The main advantage of this chloroplast region is its high amplification success rate, as confirmed here –88% of all reference samples were successfully sequenced (Table 1). This is consistent with many other studies, which show this locus typically scores the highest in this aspect [24], [48]. On the other hand *rpoC1* exhibits a slower rate of evolution than non-coding plastid regions and some plastid genes such as *matK* [53]. In this study, roughly half (45%) of all root samples yielded species level identifications and 37.5% yielded genus level identifications for rpoC1 (Fig. 2). The relatively low number of species level identifications is probably due to identical sequences for different species. Such cases would probably increase in frequency if the reference database were larger and contained more species and more diverse genera.

#### 3.1.2 PsbA-trnH

Sequencing success for this locus, although lower than that of *rpoC1*, was relatively high for reference sequences (81.4%) and moderate for root sequences (74.4%). Sequencing success was particularly low for monocots, only in 50% of the market samples and 66% of the reference samples yielded a *psbA-trnH* sequence. Discriminatory power was somewhat superior to that of *rpoC1*. Almost 60% (59.7%) of the samples that yielded a sequence could be identified to the species level and 24.2% to genus level. However, assembling the *psbA-trnH* trace files into contigs was not always straightforward, as repeats of 10 or more consecutive A’s or T’s induced Taq-polymerase errors, which made it difficult to accurately assemble the trace files. This resulted in a number of unreliable sequences that could not be used. It has been suggested that this feature of *psbA-trnH* and other non-coding regions prevent their use in future large-scale barcoding projects, in which manual editing of sequences is necessarily kept to a minimum [59]. Also, although not problematic in this study, *psbA-trnH* occurs in more than one copy in cycads [48] and in a number of sedges [54].

#### 3.1.3 ITS

ITS proved to be most useful marker for identifying samples to species level (63.8%) or genus level (29.8%) once a sequence was obtained. However, 45% of the market and reference sequences could not be used, 34% due to polymorphisms, and 11% due to fungal contamination. Fungal contamination may in this case have been caused by molds on the final dried medicinal roots or by mycorrhizal fungi that were present in the roots. Chen et al. [24] also reported a very low sequencing success rate for monocots for ITS as a whole and Gonzalez et al. [57] reported difficulties sequencing ITS in a study on Amazonian forest trees. In a recent study, the China Plant BOL Group found significantly lower levels of polymorphism and fungal contamination after sequencing a large sample of angiosperms [25]. Chen et al. [24] argue for including ITS2 as a standard barcode, but do not discuss polymorphism difficulties, and report no fungal contamination in their samples. A possible explanation for this is that the study uses leaf samples from freshly collected plant material of plants known to be used in Traditional Chinese Medicine as opposed to the processed medicinal products themselves. Their arguments to include a marker from the nuclear genome are legitimate, but we find that polymorphism and fungal contamination (particularly for root material) do cause problems in using ITS as a marker for DNA barcoding.

#### 3.1.4 BLAST

BLAST in combination with species distribution data as well as critical evaluation of the presence or absence of related species in GenBank was the most successful way to identify the market samples (Fig. 2). Several other studies also indicate that BLAST outperforms other methods like DNABAR, ATIM, Blastclust, neighbor-joining trees, and PWG-distance, the distance method adopted by the CBOL Plant Workgroup [25], [48], [52].

#### 3.1.5 RAxML

The tree-based method was relatively successful for the identification of market samples using *rpoC1* (51.3% species level identification), which is a coding region that could be readily aligned using MAFFT. The species level identification frequency for ITS was also relatively high, 48.9%. *PsbA-trnH* sequences were more difficult to identify using MAFFT and RAxML, 29%. A possible explanation for the difference in identification success between ITS and *psbA-trnH* is that the highly conserved 5.8S coding region in ITS facilitated the alignment. Also, the ITS dataset contained roughly one third more sequences than the *psbA-trnH* dataset, which might have played a role in the alignment process. A clear advantage of tree-based methods is the branch lengths, which provide a visual representation of sequence divergence. The relative success of the coding region in applying tree based methods supports the idea of using coding plastid regions as universal barcoding markers.

#### 3.1.6 Blastclust

The Blastclust analyses resulted in many unidentified samples for all markers that either belonged to clusters containing many different reference sequences or to clusters that contained only query sequence (Data S7, S8, S9). Adjusting the similarity threshold had no effect on the number of identifications, probably because different lineages have different evolutionary rates and no single threshold could fit a dataset containing many unrelated taxa, especially if there is no clear distinction between inter- and intraspecific variation.

#### 3.1.7 Role of taxonomic group

Nineteen of the 83 market samples (23%) yielded a sequence for only one of the markers, of which twelve were *rpoC1* sequences, four *psbA-trnH*, and three ITS. Of these samples one was a basal angiosperm (*Aristolochia*), ten were monocots and 8 were eudicots. This represents all the basal angiosperms, 77% of the monocots, and 12% of the eudicots.

The sequencing success for all markers was clearly higher for eudicots than for monocots (and basal angiosperms) for both market and reference samples (Table 1). This could be due to primer fit problems, secondary metabolites or differences in how well the DNA in these groups tolerate long term storage as either herbarium vouchers or dried medicinal roots.

Eudicots were on average most successfully identified using ITS (63.8% resp. 29.8% to species and genus level) after correction for the number of sequences that were obtained. Species level identification of eudicots was least frequent using *rpoC1* (48.3%). Within the eudicots the Apiaceae could be identified to species level twice as often as the Asteraceae despite a higher sequencing success for the Asteraceae. Species level identification was higher for Apiaceae than for Asteraceae for all three markers. Caryophyllaceae could either be identified to species level (in the cases of *Corrigiola* and *Silene*, the latter being due to the large number of ITS sequences for this group available in GenBank) or only to family level, showing that even within one family the evolutionary rates can differ enough to cause considerable variation in species identification success using molecular data.

All *rpoC1* monocot sequences could be identified to either species or genus level (77.8% resp. 22.2%), whereas only 50%, resp. 16.7% of the monocot *psbA-trnH* sequences could be identified to the species and genus level. Only two ITS monocot query sequences were obtained. It is noteworthy that six of the eight monocot market samples were shown to belong to the same species, *Dioscorea communis* (L.) Caddick & Wilkin.

The combined analyses did not show improved species level identification as compared to the individually analyzed markers even after we corrected for the missing query sequences (Data S1). This is in part due to the limited reference dataset that was used, but in the individual analyses identification success can often be traced back to one or two specific marker(s) whereas the other marker(s) yielded identical sequences for several species or even genera.

Our study shows a somewhat lower species level identification success-rate than several other studies that use the same markers (Table 3). This can in part be explained by the nature of the market samples. Sequencing failure for many of the market samples may be due to post-harvest processing resulting in DNA degradation, such as drying at high temperatures, slow drying under moist conditions or storage in alcohol. Another study targeting medicinal products reports similar difficulties obtaining sequence data from degraded samples [43]. Also in contrast to most studies testing the efficacy of molecular identification of plant material our reference database presumably consisted of sequences obtained from different populations than those of the query sequences, an approach that we deem realistic since a global barcoding database would inevitably only contain samples from a fraction of the populations of any given species.

**Table 3:** Overview of species level identification success (%).

	rpoC1	psbA-trnH	ITS
Burgess et al. 2011	54%	63%	–
CBOL, 2009	43%	69%	–
Chen et al, 2010	–	63%	86% (ITS2)
China Plant BOL Group, 2011	–	45%	67%
Fazekas et al., 2008	27%	59%	–
Gonzalez et al., 2009	53%	66%	80%
Kress & Erickson 2007	50%	78%	27% (ITS1)
Lahaye et al., 2008 (ML)	34%	72%	–
Muellner et al., 2011	0%	–	67%
Newmaster et al., 2007	0%	66%	–
Piredda et al., 2011	48%	73%	–
Sass et al., 2007	46%	–	81%
Starr et al., 2009	13%	44%	–
This study	45%	45%	36%

### 3.2 Ethnobotanical and Environmental Implications

Overall we found that 18% of the samples were misidentified in the pharmacopeia. The apparent discrepancy between the barcoding identifications and the vernacular names can largely be explained by the lack of a one-to-one correspondence between the vernacular names of plants (or plant products) and biological species. This phenomenon is a feature of virtually all folk classifications systems of living organisms [60]. However adulteration and misidentification play a major role as well.

### 3.3 Taxonomic Under-differentiation and Product Qualities

Nineteen samples analysed belonging to five plant products turn out to be species complexes. That is groups of species for which the same vernacular name is used. This appears to be due to taxonomic under-differentiation, which is failure to distinguish between closely related species. In some instances, the species identification for a particular root sample seems to correlate with the “quality” assigned to the root product by the herbalist. The most clear-cut case is *’ud-mserser*, of which the samples designated as the highest in quality were identified as *Daucus crinitus* Desf. (Apiaceae), whereas those designated as secondary quality were found to correspond to closely related *Thapsia* spp. (Apiaceae) [61], [62] (Table 2). Another example of under-differentiation is *nnjem* that is hypothesized to be *Cynodon dactylon* (L.) Pers. in the pharmacopoeia [3], but is found to include other grasses as well.

The various types of *sargina* (6 samples tested, see Table 3) constitute another species complex consisting of plants that belong to the carnation family (Caryophyllaceae), although here it is less clear how the types actually relate to biological entities, if they do at all. In all of these examples, the herbalists treat the species as subtypes of the same vernacular name suggesting that they are believed to share the same medicinal properties and are used to treat the same ailments.

### 3.4 Taxonomic Over-differentiation

Taxonomic over-differentiation is where one biological species is referred to by several vernacular names. For example, *frifra*, *bouzfour*, *terta* and *zziyata* were all identified as *Kundmannia sicula* DC. (Apiaceae) in at least one of the samples analysed. The most common vernacular for this species is *zziyata* according to Bellakhdar [3], while *frifra* and *bouzfour* usually refer to other members of the family [3]. The latter two cases might therefore have resulted from a misidentification by the collector. *O*n the other hand, *terta,* normally applies to the unrelated *Withania frutescens* (L.) Pauquy (Solanaceae), which in the wild is very unlikely to be confused with any of the other three species. This is more likely error on the part of the herbalist due to a mix-up of similar-looking prepared root products. *Silene* was either sold as *sargina* or as *tigigest*, but it should be noted that these names do probably refer to two not very similar looking species of *Silene* and might in fact not represent a case of taxonomic over-differentiation. *Echinops* was found to be sold as *taskra*, *besbas* and *horsef*. Only *taskra* is mentioned as a vernacular name for *Echinops* by Bellakhdar [3]. The other two product names usually refer to *Cynara* (*horsef*) or to *Foeniculum* (or possibly *Anthum foeniculoides*, cf. Data S9) in the case of *besbas* and *Echinops* seems to be popular as an adulterant for these products. The names *bougoudz* and *ndkhir* are both in use for *Dioscorea communis* a plant that is new for the Moroccan traditional pharmacopoeia. In total taxonomic over-differentiation was inferred to affect 22 samples belonging to roughly one-third (11) of the products.

### 3.5 Adulteration, Misidentification, and Toxicity

The trade in medicinal plants provides the main source of income for herbalists, and economic constraints may provide incentive for herbalists to substitute cheaper and more readily available species for rare ingredients, misleadingly selling them under the same name. Such cases of deliberate adulteration of coveted ingredients are often difficult to distinguish from cases of under or over-differentiation or misidentification. Many of the cases mentioned in the previous sections could have occurred either inadvertently (by misidentification), or purposefully.

A clear example of possible adulteration is the sample of *bukbuka*, which translates as *Colchicum autumnale* L. [3]. This plant has traditionally been used to treat acute arthritis and renal disorders [63], but Bellakhdar [3] states that it is no longer traded in Morocco owing to its extreme toxicity. Perhaps unsurprisingly, molecular identification showed the vernacular name specified by the herbalist to be misleading. Instead the sample was identified as *Bunium* sp. (for which *bukbuka* does not apply), a plant with similar bulbous underground parts, but non-toxic and entirely unrelated to *Colchicum*. If Bellakhdar’s note that *Colchicum* is no longer used in the Moroccan pharmacopoeia is correct, then the usage of the name *bukbuka* is probably intentionally deceptive. Other cases of adulteration or misidentification comprise both samples of *l-harmel* which instead of *harmala* L. were identified as *Carlina brachylepis* (Batt.) Meusel & Kästner, and a species of grape (*Vitis* sp.) and two samples of *’aqirqarha* that were identified as species of *Catananche* instead of *Anacyclus*. *’Aqirqarha* is a relatively expensive product and adulteration is therefore profitable.

In total eight samples belonging to six different products were probably adulterated, or at least misidentified. Adulteration and misidentification issues raise concerns of potentially toxic plants being sold to the consumers, sometimes without the herbalist being aware of it. However, two of the three products, which are known to be highly toxic (*bukbuka* and *l-harmel*) are clearly being replaced by less harmful plants. Only *Carlina gummifera* (L.) Less. is still being sold regularly as *addad*.

Another plant that raises public health concerns is *Arundo donax* L., a giant reed that has shown potential for use in phytoremediation of soils with high concentrations of arsenic, cadmium and lead [64]. Significantly elevated concentrations of heavy metals were found in the roots of *A. donax* grown on polluted soils [64], [65]. Elevated heavy metal concentrations might be a concern when *A. donax* roots are consumed for medicinal purposes, depending on where the plants are collected.

### 3.6 Conservation Issues

Several endemic plants are commercialized as medicinal roots (Data S10), like for example *Acacia gummifera* Willd., *Silene mentagensis* Coss., and possibly *Anethum foeniculoides* Maire & Wilczek. Endemic plants are not necessarily rare, but they could quickly become critically endangered if they are harvested in an unsustainable way. A number of products that could be identified to genus level belong to genera that contain rare or very rare species. For example half of the species of *Armeria* occurring in Morocco are rare and locally or regionally endemic. Additional field studies together with the people collecting these plants combined with a more taxon-specific barcoding approach could give insight into whether these endangered species enter the markets as well and if the plant collectors are aware of the differences in morphology and abundance between these species. The vast majority of the roots that are sold in Marrakech belong to species that are not threatened and that are common, also outside Morocco. Nevertheless, the high level of adulteration may indicate that there are species that are locally overexploited or endangered.

### 3.7 Conclusions

Roughly one fifth of the market samples that were analyzed proved to be something other than what was hypothesized on the basis of the Moroccan pharmacopoeia. There seems to be a trend towards toxic plants being replaced by species that are less dangerous. The analyses showed that several endemic and possibly also endangered plants are being commercialized in Marrakech. Adulteration is common and may indicate that the original products are becoming locally endangered. Nevertheless the majority of the medicinal roots that are sold belong to species that are common, and not known to be endangered.

Sequencing success was highest for *rpoC1* and lowest for ITS (Table 1), mainly due to polymorphism, but also due to fungal contamination. Eudicot samples yielded a higher sequencing success than monocots and basal angiosperms. Identification success was highest using BLAST combined with data on species distribution and information on presence or absence of species in the reference database. Tree-based identification, after alignment using MAFFT, was very successful for coding *rpoC1*, moderately successful for ITS and had low success for *psbA-trnH* due to alignment problems. Identification success for each marker depended on taxonomic group.

The identification success in our study is somewhat lower than in several other studies that involved testing the efficacy of molecular identification on the basis of one large dataset [32], [50] or by using query sequences from the same populations as the reference sequences [57]. This is probably due to a combination of high intraspecific variation, and low number of sequences per species in the reference datasets. A significant conclusion from our results is that unknown samples are more difficult to identify than suggested, especially if the reference sequences were obtained from different populations than the unknown material, even when the reference samples were collected in the same country. A global barcoding database should therefore contain a large number of sequences from different populations of the same species to ensure that the reference sequences characterize the species throughout its distributional range.

Although molecular identification often fails to assign individuals to species our results demonstrate that it is a helpful tool in providing clues for identifying medicinal plant products that lack morphological features for species identification.

## Materials and Methods

### 5.1 Market Samples

A total of 111 market samples of medicinal roots were bought from a total of 10 herbalists in central Marrakech. 96 of these samples were initially collected in October and November 2007, and additional samples of 15 products that proved to be difficult to sequence were collected in November 2008. All samples were stored at the herbarium of the Natural History Museum Marrakech and at Uppsala University’s herbarium (UPS). The vernacular name for each sample as communicated by the herbalist was recorded, along with the herbalist’s name and the place and date of purchase. In most cases several samples were collected per vernacular name, resulting in the collection’s comprising 37 different medicinal plant products (Table 2, Data S1). Some products are further divided by the herbalists into subtypes specified by modifiers placed after the main noun (e.g. *sargina lmsouwsa* vs. *sargina rrahmania*). Putative scientific names have been assigned to the material based on the Moroccan vernacular names, using the most recent herbal pharmacopoeia of Morocco [3]. All roots were purchased as single products to avoid mixtures of different plants.

### 5.2 Reference Database

Reference species were selected based on the putative scientific names of the 37 medicinal plant products. Species known to occur in Morocco were selected according to the Flore practique du Maroc [66], [67], Catalogue des plantes vasculaires du nord du Maroc [68], [69], Catalogue des plantes vasculaires rares, menacées ou endémiques du Maroc [70], and Flore vasculaire du Maroc [71], [72], as this is the main origin for medicinal roots traded in Marrakech [4]. All genera considered candidates for the identity of a certain market sample were comprehensively sampled, while larger genera with 7 or more species were sampled with up to three or four species (Data S2).

The reference database was complemented for market samples that could not be identified using the selection process described above by sequencing the nuclear ITS region. These ITS sequences were then queried against GenBank’s nr-database using the Megablast algorithm with default parameters. The highest-scoring hits from these queries were used as preliminary identifications to select additional reference material (Data S2).

In total, the reference database consisted of plant material from 131 herbarium specimens kept at the Reading University Herbarium (RNG), UK. Most of these voucher specimens were collected in Morocco (Data S3).

### 5.3 DNA Extraction

Root material was extracted using a slightly modified version of the Carlson/Yoon DNA isolation procedure [73]. About 2 g of each sample was fragmented into coarse grains, if necessary using a scalpel. The sample fragments were transferred to a mortar and dry-ground at room temperature with sterile grinding sand until homogenized. No more than 500 µg of the ground material was transferred to a 2 ml microfuge tube after which the regular protocol was followed.

Total DNA of leaf material of the reference samples was extracted and purified in the same way as for the market samples, but using a Mini-Beadbeater (BioSpec Products) instead of manual grinding: ca. 0.02 g of plant material was combined with silica beads, 750 µl of CTAB (hexadecyl trimethyl ammonium bromide) and 20 µl mercaptoethanol in a 2 ml tube. The tube was put into the Mini-Beadbeater and shaken for 40 seconds or more, and then incubated at 65°C for 45 min, intermittently mixed by inverting.

Each total DNA extract was further purified using the GE Illustra GFX™ PCR DNA and Gel Band Purification Kit following the manufacturer’s protocol (GE Healthcare).

### 5.4 PCR and Sequencing

Barcoding loci and primers were selected from the Royal Botanic Gardens Kew Phase 2 Protocols and Update on plant DNA barcoding [74]. These consisted of ITS primers *ITS-4* [75] and *ITS-5* [76], matK primers, *matK-2.1a* and *matK-5* [74], *rpoC1* primers, *rpoC1-2* and *rpoC1-4* [74], and *psbA-trnH* primers, *psbA* and *trnH* [77]. PCR amplification of, ITS, *matK*, *rpoC1* and *psbA-trnH* was done on purified total DNA from all reference and market samples.

PCR amplification of purified total DNA was performed in 200 µl reaction tubes with a total volume of 50 µl. Each tube contained a mixture of 5 µl reaction buffer (ABgene, 10x), 3 µl MgCl2 (25 mM), 1 µl dNTP’s (10 µM), 0.25 µl Taq-polymerase (ABgene; 5 U/µl), 0.25 µl BSA (Roche Diagnostics), 12.5 µl of each primer (2 mM) and 1 µl template DNA. The PCR conditions were as follows for the plastid markers: an initial 2 min of denaturation at 94°C followed by 38 cycles of 30 sec of denaturation at 94°C, 40 sec annealing at 53°C, and 40 sec elongation at 72°C ending with an additional elongation of 5 min at 72°C. The PCR-programs used for ITS was: an initial 5 min of denaturation at 98°C followed by 35 cycles of 30 sec of denaturation at 98°C, 1 min annealing at 55°C, and a 1 min elongation at 72°C ending with an additional elongation of 10 min at 72°C resp. an initial 2 min of denaturation at 98°C followed by 35 cycles of 10 sec of denaturation at 98°C, 1 min annealing at 60°C, and a 1 min elongation at 72°C ending with an additional elongation of 8 min at 72°C.

Following the PCR, we checked for PCR product by running 5 µl of sample with 2 µl of loading buffer on a 1% agarose gel in TAE buffer. The gel was then stained in a bath with 1% ethidiumbromide and the fragments were visualized using UV-light.

Sequencing was performed by Macrogen Inc. (Seoul, South Korea) on an ABI3730XL automated sequencer (Applied Biosystems). The same primers used in PCR amplification were also used for the sequencing reactions. Trace files were aligned with the programs Gap4 and Pregap4 [78], both modules in the Staden package [79].

### 5.5 Data Analyses

All reference sequences were submitted to GenBank. NCBI’s web-based megablast algorithm using the default settings was then used to identify the query sequences. Each identification was made manually taking E-value, maximum identity, number of closely related species represented in the database, as well as distribution of the plant(s) in question into consideration.

All sequences that yielded an e-value of 0.0 in the BLAST searches were then downloaded from GenBank in fasta-format to create an extended reference database for each marker. Sequences that were longer than 700 bp (plastid markers), resp. 800 bp (ITS) and sequences that had more than 5% unspecified nucleotides (Ns) were removed using BioPerl [80]. The query sequences were then added to the files and orientation of the sequences in each file was subsequently checked to make sure no reverse-complements were used.

Blastclust analyses [49] were done on the MPI Bioinformatics Toolkit webserver [81] for each dataset using a 98% similarity threshold for the non-coding markers (*psbA-trnH* and ITS) and a 100% similarity threshold for rpoC1 as well as a 90% minimum length coverage for all three datasets. Query sequences were identified on the basis of the reference sequences that they formed a cluster with. Similarity thresholds were determined using pairwise analysis in SpeciesIdentifier v. 1.7.8 [36].

In addition to these two alignment free methods, all three datasets were aligned using MAFFT [82] and phylograms were constructed using RAxML version 7.2.8 [83], [84] under the GTRGAMMA model with 1000 bootstrap replicates under the GTRCAT model on the Cipres Science Gateway [85]. All three phylograms were visualized using Dendroscope [86] (Data S4, S5, S6). The query sequences were identified to the species level as described in Meier et al. [36] (i.e. only if they belonged to a species specific clade, but not if the query sequence was sister to a species-specific clade) with the exception that branch lengths were taken into account so that query sequences that were identical to a sequence of a certain species in the reference database with which they formed a clade were deemed identified to the species level. Other sequences were identified to either genus or family level if they were clustered at least one node into a clade consisting of sequences from only a certain genus or family. Support values were not taken into account in the identifications.

Blastclust and RAxML analyses were performed on the combined datasets using only the reference data generated in this study. A combined data analysis that also includes GenBank data would have been ideal, but was not feasible since GenBank records often lack information on the voucher specimen, hence making it impossible to combine the extended reference databases for the different markers.

The final identification of each product was done on a case-to-case basis using the outcome of the three methods for each of the three markers (Data S3, S10) and taking into account when reference sequences from a certain species were present in one or two datasets but not the other(s).

## Supporting Information

Data S1 Table with market samples, identifications, and GenBank accession numbers.
(XLSX)

Data S2 Reference samples and GenBank accession numbers.
(DOCX)

Data S3 Map with collection sites of specimens that were used for the reference database.
(TIFF)

Data S4 RAxML phylogram of the *rpoC1* extended reference dataset plus the market *rpoC1* sequences.
(PDF)

Data S5 RAxML phylogram of the *psbA-trnH* extended reference dataset plus the market *psbA-trnH* sequences.
(PDF)

Data S6 RAxML phylogram of the ITS extended reference dataset plus the market ITS sequences.
(PDF)

Data S7 Blastclust output *rpoC1.*
(TXT)

Data S8 Blastclust output *psbA-trnH.*
(TXT)

Data S9 Blastclust output ITS.
(TXT)

Data S10 Background information per product vernacular name on putative scientific names, barcode marker sequence matching, and species identification. Distribution data and conservation status is included for all identified species.
(PDF)
