# Supplementary material for: Molecular Identification of Commercialized Medicinal Plants in Southern Morocco
Source: PLoS One. 2012 Jun 27;7(6):e39459. doi: 10.1371/journal.pone.0039459 (PMC3384669; doi:10.1371/journal.pone.0039459)
Supplement: Data S2 — Reference samples and GenBank accession numbers. (DOCX) [file pone.0039459.s002.docx]

| **Supplemental data S2.** Reference vouchers and Genbank accession numbers for the amplified loci | | | | | | |
| --- | --- | --- | --- | --- | --- | --- |
| **Species** | **Collection** | **matK** | **rpoC1** | **psbA** | **ITS** |  |
| *Acacia gummifera* Willd. | *Aparaicio, Rowe & Silvestre* s.n. |  | HE603096 | HE602486 | HE602459 |  |
| *Acacia tortilis* subsp. *raddiana* (Savi) Brenan | *S.L. Jury* 19064 |  | HE603088 | HE602480 | |  |
| *Ammoides pusilla* Breistr. | *M. Ait Lafkih & al*. 245 |  | HE603090 | HE602482 | HE602454 |  |
| *Anacyclus clavatus* (Desf.) Pers. | *J.M. Montserrat.*2043/94 |  | EU531594 | | HE602384 |  |
| *Anacyclus homogamus* (Maire) Humphries | *S.L. Jury & R. Shkw*a 20904 |  | EU531595 | EU531693 | HE602385 |  |
| *Anacyclus pyrethrum* L. | *S.L. Jury* 17869 |  | EU531592 | EU531691 | HE602382 |  |
| *Anacyclus radiatus* Loisel. | *S.L. Jury* 18550 |  | EU531593 | EU531692 | HE602383 |  |
| *Anethum foeniculoides* Maire & Wilczek | *S.L. Jury & T.M. Upson* 20571 |  | HE603091 | HE602483 | HE602455 |  |
| *Aristolochia baetica* L. | *J.M. Montserrat & B. Valdés* 2002 | EU531669 | EU531588 | HE659552 | |  |
| *Aristolochia fontanesii* Boiss. & Reut. | *Davis* 51947 | EU531668 | EU531587 | HE659551 | |  |
| *Aristolochia paucinervis* Pomel | *M. Ait Lafkih & al.* 242 | EU531670 | EU531589 | HE659553 | |  |
| *Aristolochia pistolochia* L. | *S.L. Jury* 16506 | EU531671 | EU531590 | HE659554 | |  |
| *Armeria atlantica* Pomel | *S.L. Jury* 18964 |  | HE659534 | EU531718 | HE602421 |  |
| *Armeria choulettiana* Pomel | *S.L. Jury & R. Shkwa* 20912 |  | HE659535 | HE659572 | HE602422 |  |
| *Armeria simplex* Pomel | *E. Bayón, C. Oberprieler, R. Vogt* s.n. |  |  |  | HE602461 |  |
| *Arundo donax* L. | *S.L. Jury* 17806 |  |  | EU531719 | HE602423 |  |
| *Arundo plinii* Turra | *Husain* 158 |  |  | EU531720 | HE602424 |  |
| *Asparagus acutifolius* L. | *OPTIMA ITER V.* 1250 |  |  | HE659566 | |  |
| *Asparagus albus* L. | *S.L. Jury* 12557 |  | HE659530 | EU531712 | |  |
| *Asparagus altissimus* Munby | *S.L. Jury & T.M. Upson* 20506 | EU531679 | EU531623 | | HE602411 |  |
| *Asparagus aphyllus* L. | *OPTIMA ITER V* 14 |  | HE659529 | HE659565 | HE602412 |  |
| *Asphodelus macrocarpus* ssp. *rubescens* Z. Díaz & Valdés | *M.A. Mateos & J.M. Montserrat* 5869/2 |  |  |  |  |  |
| *Astragalus armatus* Willd. | *A. Romo*  R-8883/5 |  | EU531616 | HE659563 | HE602405 |  |
| *Bryonia dioica* Jacq. | *S.L. Jury & T.M. Upson* 20437 |  | HE659527 | EU531705 | HE602401 |  |
| *Bunium alpinum* ssp. *atlanticum* Maire | *OPTIMA ITER V* 1064 |  | HE603083 | HE602474 | HE602447 |  |
| *Bunium bulbocastanum* L. | *J.M. Montserrat & B. Valdés* s.n. |  | HE603101 | HE602489 | HE602464 |  |
| *Bunium incrassatum* (Boiss.) Amo | *Davis* 52337 |  | HE603100 | HE602488 | HE602463 |  |
| *Bunium pachypodum* P.W. Ball | *M.A. Mateos & J.M. Montserrat* 5835/3 |  | HE603099 | HE602487 | HE602462 |  |
| *Carlina brachylepis* (Batt.) Meusel & Kästner | *A.J.K. Griffiths* 18069 | EU531677 | EU531608 | EU531703 | HE602398 |  |
| *Carlina gummifera* (L.) Less. | *S.L. Jury* 12348 |  | EU531591 | HE659555 | HE602381 |  |
| *Carlina involucrata* Poir. | *P. Kennedy* s.n. |  | HE603097 | |  |  |
| *Carlina lanata* L. | *S.L. Jury* 15064 |  | HE603081 | HE602472 | HE602446 |  |
| *Carlina macrophylla* (Desf.) DC. | *S.L. Jury* 11909 | EU531678 | EU531609 | EU531704 | HE602399 |  |
| *Carthamus pinnatus* Desf. | *A. Kool* 1018 (UPS) |  | HE603107 | HE602494 | HE602470 |  |
| *Catananche arenaria* L. | *S.L. Jury & T.M. Upson* 20689 | EU531674 | EU531598 | EU531695 | HE602388 |  |
| *Catananche caerulea* L. | *S.L. Jury* 11396 | EU531675 | HE659524 | EU531696 | HE602389 |  |
| *Catananche caespitosa* Desf. | *S.L. Jury* 18111 | EU531672 | EU531596 | HE659556 | HE602386 |  |
| *Catananche lutea* L. | *R. Vogt* 12348 | EU531673 | EU531597 | EU531694 | HE602387 |  |
| *Catananche montana* Coss. & Durieu | *M.A. Mateos* 7039/95 | EU531676 | HE659525 | HE659557 | |  |
| *Ceratolimon feei* (Girard) M.B.Crespo & Lledó | *S.L. Jury* 19168 | EU531681 | EU531637 | EU531717 | |  |
| *Corrigiola litoralis* subsp. *litoralis* L. | *Källsten* s.n. (UPS) |  | EU531661 | |  |  |
| *Corrigiola litoralis* subsp. *litoralis* L. | *S.L. Jury* 18530 |  | HE603087 | HE602479 | HE602452 |  |
| *Corrigiola litoralis* subsp. *telephiifolia* Pourr. | *S.L. Jury* 19483 |  | HE603089 | HE602481 | HE602453 |  |
| *Cynara baetica* (Spreng.) Pau | *S.L. Jury* 11233 |  | EU531601 | EU531698 | HE602391 |  |
| *Cynara humilis* L. | *S.L. Jury* 19316 |  | EU531602 | EU531699 | HE602392 |  |
| *Cynodon dactylon* (L.) Pers. | *M.A. Mateos, A. Ortega & F.J. Pina* 7291 |  | HE659536 | EU531721 | HE602425 |  |
| *Cyperus longus* L. | *S.L. Jury* 11493 |  | EU531611 | |  |  |
| *Cyperus rotundus* L. | *S.L. Jury* 12712 |  | EU531612 | |  |  |
| *Daucus aureus* Desf. | *Reading Univ./B. M. Exped.* 1076 |  | EU531584 | EU531688 | HE602378 |  |
| *Daucus carota* L. | *Jury, S.L.* 17848 |  | EU531582 | EU531686 | HE602376 |  |
| *Daucus crinitus* Desf. | *M. Ait Lafkih & al.* 70 |  | EU531580 | EU531684 | HE602443 |  |
| *Daucus durieua* Lange | *M.J. Díez* 3482/94 |  | EU531583 | EU531687 | HE602377 |  |
| *Daucus muricatus* (L.) L. | *S.L. Jury* 16748 |  | EU531585 | EU531689 | HE602379 |  |
| *Daucus setifolius* Desf. | *S.L. Jury* 17514 |  | EU531581 | EU531685 | HE602375 |  |
| *Dioscorea communis* (L.) Caddick & Willkin. | *C. Harrouni, S.L. Jury & T.M. Upson* 20616 |  | HE603093 | |  |  |
| *Drimia fugax* (Moris) Stearn |  |  | EU531659 | |  |  |
| *Drimia maritima* (L.) Stearn | *S.L. Jury* 12554 |  | EU531628 | |  |  |
| *Drimia maura* (Maire) J.C.Manning & Goldblatt | *S.L. Jury* 9030 |  | EU531630 | HE659568 | HE602414 |  |
| *Drimia undata* Stearn | *S.L. Jury* 13275 |  | EU531629 | HE659567 | HE602413 |  |
| *Echinops fontqueri* Pau | *J. Lambinon & G. van den Sande* 94/Ma/454 |  | HE603098 | |  |  |
| *Echinops spinosissimus* Turra | *S.L. Jury* 18328 |  | EU531606 | HE659559 | HE602460 |  |
| *Elaeoselinum meoides* W.D.J.Koch ex DC. | *S.L. Jury & T.M. Upson* 20572 |  | HE603092 | HE602484 | HE602456 |  |
| *Elymus repens* (L.) Gould | *S.L. Jury* 17625 |  |  | EU531722 | HE602426 |  |
| *Erophaca baetica* subsp. *baetica* (L.) Boiss. | *S.L. Jury & R. Shkwa* 20966 |  | EU531615 | | HE602404 |  |
| *Eryngium campestre* L. | *S.L. Jury* 17523 |  | HE603086 | HE602478 | HE602451 |  |
| *Eryngium ilicifolium* Lam. | *M.A. Mateos & B. Valdés* 641/93 |  | HE603103 | | HE602466 |  |
| *Eryngium tricuspidatum* L. | *S.L. Jury & R. Shkwa* 20881 |  | HE603094 | HE602485 | HE602457 |  |
| *Eryngium triquetum* Vahl | *J.A. Mejías & S. Silvestre* 308 |  | HE603095 | | HE602458 |  |
| *Ferula communis* L. | *S.L. Jury* 16346 |  | HE603085 | HE602477 | HE602450 |  |
| *Foeniculum vulgare* Miller | *S.L. Jury* 17822 | EU531667 | EU531579 | HE659550 | HE602374 |  |
| *Fraxinus angustifolia* Vahl | *S.L. Jury* 15547 |  | EU531631 | HE659569 | HE602415 |  |
| *Glycyrrhiza foetida* L. | *S.L. Jury* 14940 |  | EU531614 | EU531706 | HE602403 |  |
| *Glycyrrhiza glabra* L. | *J. Pedrol* 4493 |  | EU531613 | HE659562 | HE602402 |  |
| *Haplophyllum broussonetianum* Coss. | *S.L. Jury* 14466 |  | EU531658 | HE659547 | HE602442 |  |
| *Inula montana* L. | *R.K. Brummitt* 18721 |  | EU531607 | HE659560 | HE602397 |  |
| *Juglans regia* L. | *M.A. Mateos,* 7147/95 |  | EU531621 | HE659564 | HE602409 |  |
| *Juncus fontanesii* Gay | *S.L. Jury* 18967 |  | EU531622 | | HE602410 |  |
| *Kundmannia sicula* (L.) DC. | *J.M. Montserrat 3604/5* |  | HE603102 | HE602490 | HE602465 |  |
| *Launaea arborescens* (Batt.) Murb. | *S.L. Jury* 12745 |  | EU531600 | HE659558 | |  |
| *Limoniastrum* *guyonianum* Durieu ex Boiss. | *J. Lambinon 99/Tu/114* |  | HE659532 | HE659570 | HE602418 |  |
| *Limoniastrum monopetalum* (L.)Boiss. | *M.A. Mateos* 4825/95 |  | HE659533 | HE659571 | HE602419 |  |
| *Magydaris panacifolia* Lange | *H.J.M. Bowen* 8412 |  | HE603084 | HE602475 | HE602448 |  |
| *Mandragora officinarum* L. | *S.L. Jury* 12238 |  |  | HE659544 | |  |
| *Nerium oleander* L. | *M.A. Mateos* 7113 |  | EU531586 | EU531690 | HE602380 |  |
| *Ononis natrix* L. | *S.L. Jury & T.M. Upson* 20696 |  | EU531617 | EU531707 | HE602406 |  |
| *Ornithogalum narbonense* L. | *S.L. Jury* 19343 |  |  |  |  |  |
| *Peganum harmala* L. | *OPTIMA ITER V* 124 |  | EU531656 | |  |  |
| *Pinus halepensis* Mill. | *A.J. Carus, M.A. Mateos & F.J. Pina* 5266 |  | HE659531 | EU531714 | |  |
| *Pinus nigra* W.Arnold | *S.L. Jury* 12487 |  |  | EU531715 | HE602416 |  |
| *Plumbago europaea* L. | *OPTIMA ITER V* 1026 | EU531680 | EU531635 | EU531716 | HE602417 |  |
| *Populus alba* L. | *Ait Lafkih, M.* 713 |  | EU531647 | HE659540 | HE602432 |  |
| *Populus euphratica* Olver | *S.L. Jury* 17803 |  | EU531648 | | HE602434 |  |
| *Populus nigra* L. | *S.L. Jury* 18124 |  | EU531662 | | HE602433 |  |
| *Pulicaria mauritanica* Coss. | *S.L. Jury & T.M. Upson* 20667 |  | EU531604 | EU531702 | HE602395 |  |
| *Pulicaria odora* (L.) Rchb. | *M. Ait Lafkih & al.* 110 |  | HE659526 | EU531700 | HE602393 |  |
| *Pulicaria paludosa* Link | *S.L. Jury* 17773 |  | EU531603 | EU531701 | HE602394 |  |
| *Pulicaria undulata* (L.) C.A.Mey. | *S.L. Jury* 19074 |  | EU531605 | | HE602396 |  |
| *Quercus rotundifolia* Lam. | *A. Achhal, F. Bombardó & Romo* 6761/4 |  |  |  | HE602467 |  |
| *Retama monosperma* (L.) Boiss. | *S.L. Jury & T.M. Upson* 20671 |  | EU531618 | EU531708 | HE602407 |  |
| *Retama raetam* (Forssk.) Webb & Berth. | *S.L.Jury* 15876 |  | EU531619 | EU531709 | |  |
| *Rhaponticum acaule* DC. | *S.L. Jury* 15826 |  |  | HE602476 | HE602449 |  |
| *Rubia peregrina* L. | *S.L. Jury* 14983 |  | EU531644 | | HE602427 |  |
| *Rubia tinctorum* L. | *S.L. Jury* 19809 | EU531682 | | EU531723 | HE602444 |  |
| *Ruta angustifolia* Pers. | *S.L. Jury* 17639 |  | HE659538 | HE659575 | HE602430 |  |
| *Ruta angustifolia* Pers. | *S.L. Jury & R. Shkwa* 20963 |  | EU531646 | HE659539 | HE602431 |  |
| *Ruta chalepensis* L. | *Davis* 49757 |  | EU531645 | HE659574 | HE602429 |  |
| *Ruta montana* | *S.L. Jury* 17505 |  | HE659537 | HE659573 | HE602428 |  |
| *Saponaria glutinosa* M.Bieb. | *S.L. Jury* 17900 |  | EU531610 | HE659561 | HE602400 |  |
| *Scolymus hispanicus* L. | *S.L. Jury* 11671 |  | HE603080 | HE602471 | HE602445 |  |
| *Scorzonera caespitosa* Pomel. | *M. Ait Lafkih & al*. 26 |  | EU531599 | EU531697 | HE602390 |  |
| *Sesamum indicum* L. | *R.M. Nesbitt* 1939 |  | EU531633 | EU531713 | |  |
| *Silene filipetala* Litard. & Maire | *Reading Univ./BM. Exped.* 547 |  | HE603106 | HE602493 | HE602469 |  |
| *Silene portensis* L. | *M.A. Mateos & J.M. Montserra*t 5651/4 |  | HE603105 | HE602492 | |  |
| *Silene vulgaris* L. | *M.A. Mateos, E. Ramos & J. Villarreal* 5726/95 | | HE603104 | HE602491 | HE602468 |  |
| *Smilax aspera* L. | *S.L. Jury* 15315 |  | EU531650 | HE659543 | |  |
| *Tamarix africana* Poir. | *S.L. Jury* 16673 |  | EU531652 | | HE602438 |  |
| *Tamarix amplexicaulis* Ehrenb. | *S.L. Jury* 19081 |  | EU531655 | EU531725 | HE602440 |  |
| *Tamarix aphylla* | *S.L. Jury* 19079 |  | HE659523 | HE659545 | HE602437 |  |
| *Tamarix canariensis* Willd. | *OPTIMA ITER V* 1765 |  | EU531654 | EU531724 | |  |
| *Tamarix gallica* L. | *M.A. Mateos* 4301/94 |  | EU531653 | HE659546 | HE602439 |  |
| *Tetraena fontanesii* (Webb & Berthel.) Beier & Thulin | *S.L Jury & T.M Upson* 20493 |  | EU531657 | | HE602441 |  |
| *Thapsia garganica* L. | *P. Kennedy* s.n. |  | HE603079 | |  |  |
| *Thapsia platycarpa* Pomel | *S.L. Jury* 15837 | EU531666 | EU531578 | HE659549 | HE602373 |  |
| *Thapsia transtagana* Brot. | *S.L. Jury* 16325 | EU531665 | EU531577 | HE659548 | HE602372 |  |
| *Thapsia villosa* L. | *J. Lambinon* 94/Ma/350 | EU531664 | EU531576 | EU531683 | HE602371 |  |
| *Trigonella foenum-graecum* L. | *S.L. Jury* 13715 |  | EU531620 | EU531710 | |  |
| *Trigonella gladiata* M.Bieb. | *OPTIMA ITER V* 459 |  | HE659528 | EU531711 | HE602408 |  |
| *Verbascum dentifolium* Delile | *M. Ait Lafkih & al.* 104 |  | EU531649 | HE659542 | HE602436 |  |
| *Verbascum sinuatum* L. | *OPTIMA ITER V.* 1796 |  | HE659522 | HE659541 | HE602435 |  |
| *Withania frutescens* (L.) Pauquy | *A. Kool* 50 (UPS) |  | EU531660 | |  |  |
| *Ziziphus lotus* Lam. | *S.L. Jury* 15149 |  | HE603082 | HE602473 | |  |
| Unless indicated, all material is stored at RNG. Herbarium abbreviations are according to Holmgren et al, 1990. | | | | | | |
