# Supplementary figures and images for: Molecular Identification of Commercialized Medicinal Plants in Southern Morocco
Source: PLoS One. 2012 Jun 27;7(6):e39459. doi: 10.1371/journal.pone.0039459 (PMC3384669; doi:10.1371/journal.pone.0039459)

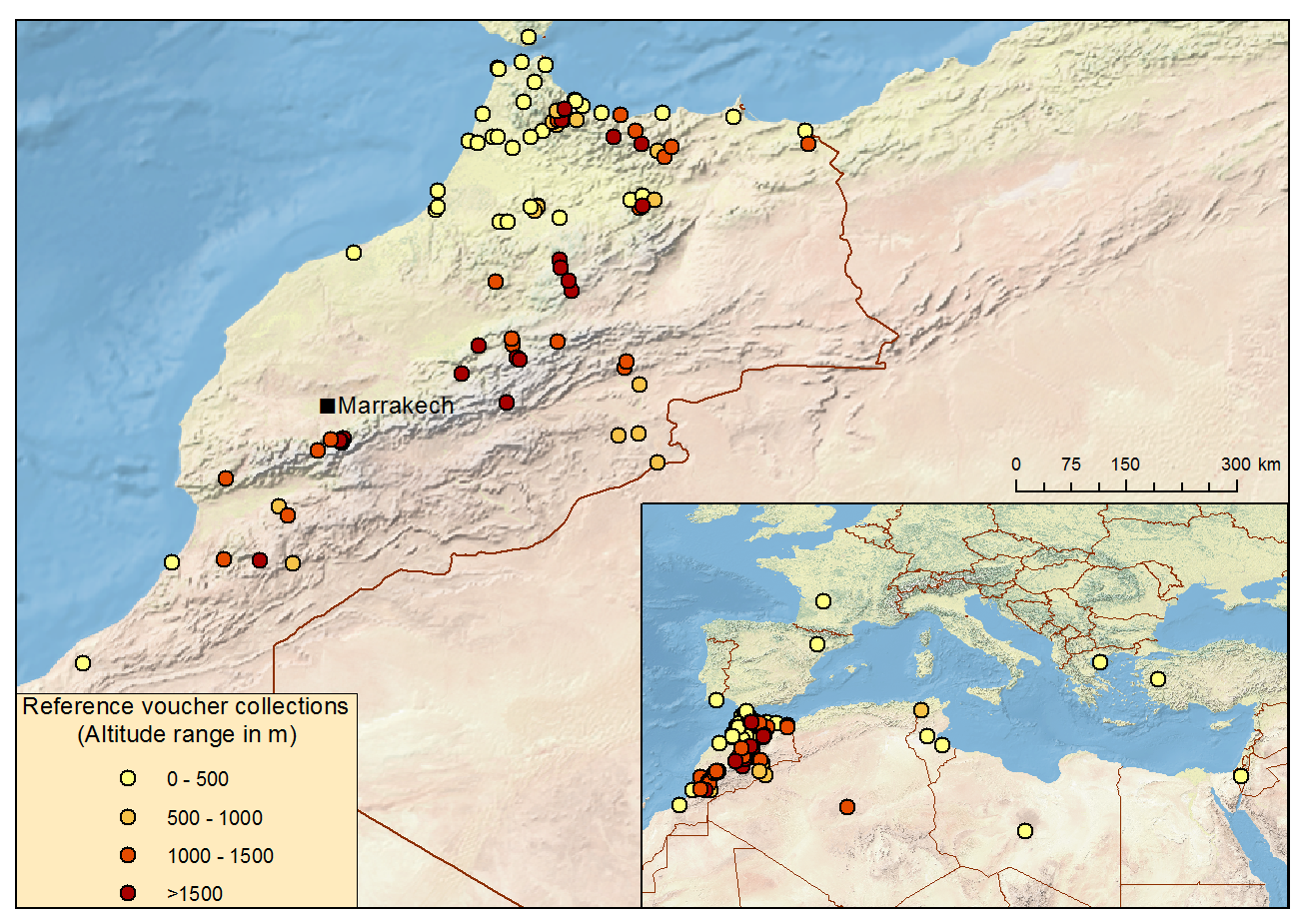

Supplement: Data S3 — Map with collection sites of specimens that were used for the reference database. (TIFF) [file pone.0039459.s003.tiff]

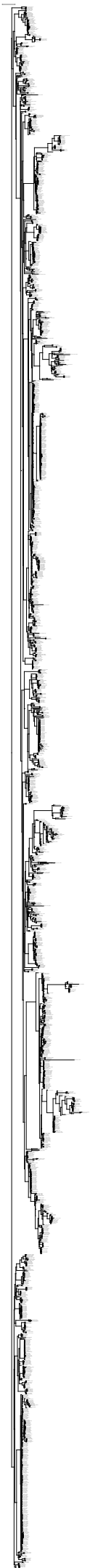

Supplement: Data S4 — RAxML phylogram of the rpoC1 extended reference dataset plus the market rpoC1 sequences. (PDF) [file pone.0039459.s004.pdf]

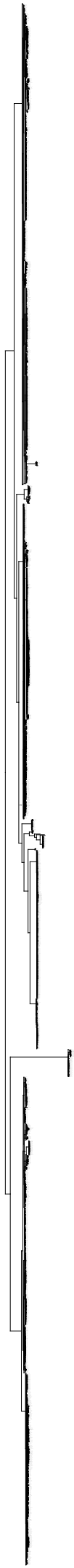

Supplement: Data S5 — RAxML phylogram of the psbA-trnH extended reference dataset plus the market psbA-trnH sequences. (PDF) [file pone.0039459.s005.pdf]
