# Supplementary material for: Molecular Identification of Commercialized Medicinal Plants in Southern Morocco
Source: PLoS One. 2012 Jun 27;7(6):e39459. doi: 10.1371/journal.pone.0039459 (PMC3384669; doi:10.1371/journal.pone.0039459)
Supplement: Data S10 — Background information per product vernacular name on putative scientific names, barcode marker sequence matching, and species identification. Distribution data and conservation status is included for all identified species. (PDF) [file pone.0039459.s010.pdf]

Supplemental data S10. Background information per product vernacular name on putative scientific names, barcode marker sequence matching, and species identification. Distribution data and conservation status is included for all identified species.

'Ansal
The vernacular name *'ansal* refers to two species of *Drimia*, *D. maritima* (L.) Stearn and *D. noctiflora* (Batt. & Trab.) Stearn (listed as *Urginea*, in Bellakhdar [1]). The market sample of *'ansal* could be confirmed to belong to either *D. undata* Stearn, *D. maura* (Maire) J.C.Manning & Goldblatt or *D. maritima*, on the basis of all three markers. *D. maura* is endemic to Morocco. The other two species are widespread and relatively common [2–6].

'Aqirqarha
'Aqirqarha is commonly traded under the Berber name *tigndizt.* A total eight market samples of *'aqirqarha* were analyzed. RpoC1 sequences were obtained from all samples, while psbA was obtained from six samples, and ITS from four. On the basis of the molecular markers five samples could be identified as *Anacyclus* sp. and three samples as *Catananche* sp. Infrageneric sequence variation was too low for most samples to identify the species, but one sample could be identified as *Catananche caespitosa* Desf. The three samples that were identified as *Catananche* sp. were bought at the *mellah*, whereas the samples identified as *Anacyclus* were all but one purchased elsewhere. *Catananche* species are not mentioned as medicinal by Bellakhdar [1], or any other online reference sources. There seems to be no relation between products described as being of high or low quality and the actual genus that is being sold. The Flore vasculaire du Maroc [3,4] lists eight species of *Anacyclus*, one of which is endemic to Morocco, *A. maroccanus* (Ball) Ball, and one is recorded as rare, *A. monanthos* (L.) Thell. Five species of *Catananche* occur in Morocco, four of which are locally common, and one is rare, *Catananche montana* Coss. & Durieu [2–6].

'Arq assus
Four different types of *'arq assus* were analyzed. One was sold as *'arq assus*, one as inland *'arq assus (lbldi)*, one as fragile *'arq assus (lhchich)*, and one as robust *'arq assus (lqash)*. All four can be identified as *Glycyrrhiza glabra* L., *G. uralensis* Fisch., or *G. inflata* Batalin (since there is virtually no sequence divergence between these species). The Flore pratique du Maroc [6] lists *G. glabra* as widely cultivated in Morocco and *G. foetida* Desf. as indigenous. The sequences of *G. foetida* for all three markers are very different from the *G. glabra* cluster, and this makes *G. glabra* the most likely candidate for the market material. However, liquorice root is widely used worldwide and it cannot be ruled out that some of the material sold by the herbalists in Marrakech is actually imported from China or elsewhere. *G. foetida* is not threatened [2–6].

'Erq wadmi
Bellakhdar [1] mentions that the name *'erq wadmi* refers to the roots of both *Armeria mauritanica* Wallr. and *A. alliacea* (Cav.) Hoffmanns. & Link. Our two samples consisted of *'erq wadmi* from the inland (*lbldi*) and foreign *'erq wadmi*

(*rroumi*). Sequence variation was low in all markers and the samples could both be identified to the genus level as *Armeria* sp. on the basis of the ITS sequences. The Flore Vasculaire du Maroc [3,4] lists eight species of *Armeria*, four of which are rare to very rare. Two of these species, *A. alpinifolia* Pau & Font Quer and *A. tingitana* Boiss. & Reut., are endemic to Morocco [2–6].

'Ud-mserser

High quality *3oud 'ud-mserser* is identified as *Daucus crinitus* Desf. without any conflict by all three markers. There is a gradual decrease in max identity between *D. crinitus* and the next-best hit, *D. carota* L., for all three markers. According to Bellakhdar [1] the name *'ud-mserser* is primarily used for *Polygonum aviculare* L., but can occasionally be applied to *D. crinitus* as well, and the latter seems to be the case here. The lower quality *'ud-mserser* is identified as *Thapsia* spp. on the basis of psbA and rpoC1. The rpoC1 sequences of *T. garganica* L., *T. platycarpa* Pomel and *T. transtagana* Brot. are identical and the only *Thapsia* psbA reference sequence available was obtained from *T. villosa* L., and shows only 95% identity with the query sequences. Unfortunately no ITS sequences were obtained from the market samples. *T. garganica* and *T. platycarpa* are not mentioned in the Flore pratique du Maroc [6], but the specimen of *T. platycarpa* that is one of the reference collections (*Jury 15837*) was collected in Morocco, close to the Algerian border. Bellakhdar [1] mentions that *T. garganica* only occurs in Morocco and Libya, and that both *T. villosa* and *T. garganica* are used medicinally, but he does not mention the name *'ud-mserser*. Pujades-Salva & Plaza-Arregui [7] mention that five species occur in Northwestern Africa: *T. transtagana, T. villosa, T. platycarpa, T. garganica*, and *T. cinerea* A.Pujadas. A phytochemical study [8] has shown significant differences in chemical constituents and fruit morphology between *T. transtagana* and *T. garganica*. The ITS reference sequences of *T. platycarpa* and *T. transtagana* differ by two substitutions, and the *T. villosa* sequence was more dissimilar. However, no sequences were obtained for the other species, and it is therefore not possible to identify this product any further than a locally occurring *Thapsia*, on the basis of our barcoding data. Neither *Daucus crinitus*, nor the species of *Thapsia* occurring in Morocco are listed as rare or endemic in Catalogue des plantes vasculaires rares, menacées ou endémiques du Maroc [2].

Addad

Addad refers to *Carlina gummifera* (L.) Less (= *Atractylis gummifera* L.) [1], a highly poisonous plants that has been the cause of many fatal poisonings, especially among children, who like the sweet flavor of the root [9], and women, who use it as an abortifacient [1]. It is also used widely in Morocco, and other countries in the Mediterranean, to induce vomiting [9]. Five samples of different subtypes of *addad* were analyzed: *addad*, *addad bjftou* (with bark), *addad lmjlaf* (without bark), *addad dkr* (male), and *addad ntwa* (female). Four samples could be confirmed to be *C. gummifera*, basis primarily on psbA and ITS sequence data. RpoC1 sequences from *C. lanata* L. and *C. gummifera* were identical. The fifth sample, *addad ntwa*, turned out to belong to another genus in Asteraceae, on the basis of its psbA sequence, but an exact match could not be found in GenBank. The *addad ntwa* psbA sequence showed

93%, resp. 94% similarity to two sequences from the genus *Rhaponticum*. *Carlina gummifera* is not considered threatened in Morocco [2].

As-susan

Our sample of *as-susan* could be confirmed to be a species of *Iris* on the basis of its rpoC1 and its psbA sequences. Bellakhdar [1] states that the name *as-susan* is used for *I.* x *germanica* L., *I.* x *florentina* L. (the latter a synonym of *I.* x *germanica*), and *I. pseudacorus* L. The Flore vasculaire du Maroc [4] list fourteen species of *Iris* for Morocco of which eight as threatened. *I.* x *germanica* is commonly cultivated in the area around Asni, south of Marrakech [10].

Bereztom

Bereztom refers to *Aristolochia longa* L. (= *A. fontanesii* Boiss. & Reut.) and *A. baetica* L. [1]. On the basis of the rpoC1 sequence, the market sample could be confirmed as a species of *Aristolochia*. Four species of *Aristolochia* occur in Morocco [3], the former are common, and the other species, *A. pistolochia* L. and *A. sempervirens* L., have unknown conservation status [2].

Besbas

Five market samples were analyzed; three were sold as wild *besbas* and two as cultivated. One wild sample was identified as *Echinops* sp., but based on the ITS sequence only (the other markers are missing). The other two wild samples can be identified (on the basis of resp. rpoC1 and both rpoC1 and psbA) as either *Anethum foeniculoides* Maire & Wilczek or *Foeniculum vulgare* Mill. Sequence variation between these two species is minimal for all three markers. The cultivated *besbas* samples yielded sequences for all three markers, and were also identified as *F. vulgare* or *A. foeniculoides*. A more detailed study on the relationship of these two species would be interesting but does not fit within the scope of this study. Bellakhdar [1] does not mention any uses of *A. foeniculoides*. *F. vulgare* is common in Morocco, but *A. foeniculoides* is assessed as rare and endangered [2].

Bid al-gul

Bellakhdar [1] lists *bid al-gul* as a vernacular name for *Mandragora autumnalis* Bertol. Our market sample could be identified as *M. officinarum* L. and not *M. autumnalis* on the basis of its psbA sequence. The Flore pratique du Maroc [6] lists only *M. autumnalis* for Morocco, but lists *M. officinarum* as a synonym (possibly pro parte as in Flora Europaea [11]). Although *M. officinarum* has priority over *M. autumnalis*, it seems appropriate to keep *M. autumnalis* and *M. officinarum* as two separate species (e.g. [11,12]). Based on our data, *M. officinarum* is commercialized in Morocco. *Mandragora* occurs widely in Morocco, and is not considered rare [6].

Bougoudz, Ndkhir

Neither *bougoudz*, nor *ndkhir* are mentioned in the Moroccan herbal pharmacopoeia [1]. Analysis of five samples of *bougoudz*, and one of *ndkhir*, yielded rpoC1 sequence for all samples, and all samples could be positively identified as *Dioscorea communis* (L.) Caddick & Willkin. In Algeria and Lebanon, *D. communis* is used against rheumatism [13,14], and in Italy it is

used in traditional herbal veterinary medicine and food [15]. Bellakhdar et al. [16] mention a plant product called *bu-goudz*, and tentatively name it *Dahlia variabilis* (Willd.) Desf. (= *Dahlia pinnata* Cav.). Boulos [17] records the use of *D. communis* in Egypt. *D. communis* is common in mountainous areas in Morocco [2–6].

Bu-zfur
According to Bellakhdar [1] *bu-zfur* is a vernacular name for *Daucus crinitus* Desf. One market sample of *bu-zfur* was analyzed, and on the basis of all three markers it could be identified as *Kundmannia sicula* (L.) DC., which is normally referred to as *zziyata* [1]. It would be necessary to obtain sequence data from several other *bu-zfur* samples to establish whether this product is *K. sicula* or if this was a one-time mix-up. *K. sicula* is common in Morocco [2–6].

Buglam sahrawi
Buglam sahrawi is a name used for *Spergularia media* (L.) C.Presl. (= *Spergularia marginata* (DC.) Kittel), a plant used to treat female infertility [1]. Sequencing success on the market sample was limited to psbA, and showed that the root indeed belongs to the family Caryophyllaceae. *S. media* is a common weed in Morocco [2–6].

Bukbuka
Bukbuka is the name for *Colchicum autumnale* L., and several other species of *Colchicum*, according to Bellakhdar [1], who also states that the plant is no longer used in traditional medicine because of its known toxicity. The market sample of *bukbuka* can be identified as a species of *Bunium* on the basis of the molecular markers. In addition, the morphology of the tuber is clearly not that of a monocot. Bellakhdar [1] lists *talgudi*, *akutar*, or *left lehla* as vernacular names for *Bunium*, but these names were not recorded during our market survey. We hypothesize that the name *bukbuka* came to be applied to *Bunium* after the herbalists stopped trading *Colchicum*. The Flore vasculaire du Maroc [3] lists three species of *Bunium*, *B. alpinum* Waldst. & Kit., *B. bulbocastanum* L. and *B. pachypodum* P.W.Ball). All three species are widely distributed in the region, and *B. alpinum* subsp. *atlanticum* Maire is probably the only endemic in Morocco [2].

Dbag
The sample of *dbag* that was analyzed yielded an ITS sequence only, and could be identified as an oak belonging to the *Quercus ilex* group [18], which corresponds with the use of the vernacular name [1]. *Quercus ilex* is common all over Morocco [2–6].

Deryas
Bellakhdar [1] suggests that the vernacular name *deryas* refers to *Thapsia*. The market sample could be confirmed to be a species in the Apiaceae family, possibly *Thapsia*, but only on the basis of a single rpoC1 sequence.

Frifra
One of the two samples of *frifra* could be positively identified as *Kundmannia sicula* (L.) DC., based on three markers, whereas the other sample yielded an

rpoC1 sequence that is 99% identical to *Foeniculum vulgare* Mill. sequences and a psbA sequence that is 94% identical to *Pimpinella aurea* DC. and *Anethum foeniculoides* Maire & Wilczek sequences, and only 93% identical to our *F. vulgare* psbA sequence. Bellakhdar [1] mentions *frifra* as a name that is used for *Magydaris panacifolia* Lange, and *zziyata* as a vernacular name for *K. sicula* (in addition to *Conium maculatum* L., *Limoniastrum guyonianum* Durieu ex Boiss., *Polygonum maritimum* L. and *Helosciadium nodiflorum* (L.) Koch (= *Apium nodiflorum* (L.) Lag.).

Fuwwa

Fuwwa refers to *Rubia peregrina* L. and *Rubia tinctorum* L. [1]. Four samples of *fuwwa* were analyzed: one sample of generic *fuwwa*, two samples of *fuwwa lfrougiuyya* i.e. from the Frouga region, southeast of Marrakech, and one sample of *fuwwa rqiqa* (*jbal nawahi mmrakch*) i.e. thin *fuwwa* from the mountains around Marrakech. The sample reportedly originating from the mountains around Marrakech, could be identified as a species of *Galium*. Two of the other three samples were doubtfully identified as *Rubia* sp. and the last one as Rubiaceae on the basis of their chloroplast sequences. The ITS sequences from these three samples showed only 90% identity with *Rubia* ITS sequences, but these were nonetheless the best hits. Sequence variation within species seems to exceed interspecific sequence variation in this group of Rubiaceae. No species of *Galium* are mentioned in the herbal pharmacopoeia [1]. There are two species of *Rubia* (*R. peregrina* and *R. tinctorum*) and 31 species of *Galium* in Morocco. The *Rubia* species are both considered common, and of the *Galium* species, fourteen are considered rare or very rare [2–6].

Fwila

Fwila refers to *Astragalus lusitanica* Lam. (= *Erophaca baetica* subsp. *baetica* (L.) Boiss.) [1]. The market sample of *fwila* could be confirmed to be *E. baetica* subsp. *baetica* on the basis of the rpoC1 and the ITS sequences. *Erophaca baetica* subsp. *baetica* is not known to be threatened [2].

Horsef

Bellakhdar lists *horsef* as *Cynara cardunculus* L., but mentions that in the Western Sahara *Echinops spinosissimus* Turra is sometimes also available under the name *horsef* with the same use [1]. Two samples of *horsef* were analyzed, one sold as regular *horsef*, and the other as foreign *horsef* (*rroumi*). The regular *horsef* sample is most probably a species of *Cynara*, as several other species of *Cynara* scored equally well in all analyses (*C. humilis* L., *C. baetica* (Spreng.) Pau, and *C. scolymus* L.). The *horsef rroumi* sample is probably a species of *Echinops*, based on rpoC1 and ITS, but the psbA sequence shows no more than 92% identity with anything. All three Cynara species are present in Morocco, *C. cardunculus* is widely cultivated, *C. baetica* and *C. tournefortii* are endemic to Morocco and the Iberian Peninsula, and the latter is considered very rare in Morocco [2–6]. Two species of *Echinops*, *E. spinossissimus* and *E. strigosus* L., occur in Morocco, and neither of them is rare [2].

L-fijel

L-fijel is a name that according to Bellakhdar [1] is used for the three species in Rutaceae, *Ruta montana* (L.) L., *R. chalepensis* L. and *Haplophyllum vermiculare* Hand.-Mazz. The studied sample could be positively identified as *R. montana* on the basis of rpoC1, as well as ITS. Other *Ruta* species has sufficiently dissimilar sequences (92% or less identity for rpoC1 and ITS) to be ruled out in identification of the market sample. *Ruta montana* is common almost everywhere in Morocco [2–6].

L-gseb
L-gseb in Arabic, or *aganim* in Berber, is a term used for *Arundo donax* L. and *Phragmites australis* (Cav.) Trin. ex Steud. [1]. Both market samples could be identified as *A. donax* on the basis of psbA alone. *Arundo donax* is a giant reed that is abundant in disturbed areas around the Mediterranean region [2–6].

L-harmel
L-harmel usually refers to *Peganum harmala* L. [1]. The seeds are widely used in Northern Africa and the Middle East for a range of purposes [19]. *P. harmala* is far from harmless, and severe neurotoxic effects have been reported after ingestion of *Peganum* seeds (e.g. [20]). The seeds are most commonly, but the roots are used to treat inflammation of the gums. *P. harmala* is common all over Morocco. The two purchased market samples of *l-harmel* are clearly not *P. harmala*. One was identified as *Carlina brachylepis* (Batt.) Meusel & Kästner based on all three markers. The other one is most probably a species of grape (*Vitis* sp.), based on rpoC1 and psbA sequence data. *Vitis* does not naturally occur in Morocco, but a closely related genus, *Ampelopsis*, does. Bellakhdar [1] does not mention *C. brachylepis*, and we were unable to find any reported uses of the species. Several uses and names are reported for grapes and raisins in Moroccan traditional medicine [1], but there are no reported uses for *Vitis vinifera* L. roots. *C. brachylepis* is common in Morocco, and *V. vinifera* is commonly cultivated [2–6].

Lghzghaz
Lghzghaz is not mentioned by Bellakhdar [1]. Sequences from the market sample for each of the three barcoding markers matched with *Carlina brachylepis* (Batt.) Meusel & Kästner. One of the *L-harmel* samples was also found to be *C. brachylepis*. It could be that *C. brachylepis* in *lghzghaz* is used as a substitute for a species that was not detected in this study. *C. brachylepis* is common in Morocco [2–6].

Lkleh
Lkleh is a vernacular name used for *Ferula communis* L. all over the Arab world [1]. The market sample could be confirmed to be *F. communis* on the basis of all three barcoding markers. *F. communis* is common in Morocco [2–6].

Luwwaya
Luwwaya is a generic term for climbing vines, including *Smilax* sp. according to Bellakhdar [1]. The market sample of *luwwaya* was identified as a species of *Smilax* based on its rpoC1 sequence. *Smilax aspera* is the only *Smilax*

species occurring in Morocco. It is common everywhere in the Mediterranean area [2–6].

Mgizla
Mgizla is a name used specifically for the roots of *Eryngium triquetrum* Vahl [1]. Two samples were analyzed, and both are certainly species of *Eryngium*, but sequence variation was too low to identify them to species level. The Flore pratique du Maroc [6] lists seventeen species of *Eryngium*. *Eryngium* consists of approximately 250 species, of which many are hypothesized to be the result of rapid radiations, and sequence variation in the genus is known to be low [21,22].

Nnjem
Nnjem is reported by Bellakhdar [1] as either *Cynodon dactylon* (L.) Pers. or *Agropyron repens* (L.) P. Beauv. (= *Elymus repens* (L.) Gould). Two market samples were analyzed; one was sold as irrigated *nnjem* (*lmawi*), and the other one as dry *nnjem* (*lbori*). Both samples could be confirmed to be grasses on the basis of respectively, a psbA, and a psbA and ITS barcoding marker. The *nnjem lbori* psbA sequence was most similar to the reference *C. dactylon* sequence (99%), but several other grass genera showed 98% identity. The psbA sequence obtained from *nnjem lwami* was 99% identical to the psbA sequences from several *Panicum* species, but also to sequences from *Setaria, Pennisetum* and *Zulogea*. The psbA sequence is 98% identical to the *Cynodon dactylon* psbA reference sequence and psbA sequences from 24 other grass genera and one, possibly misidentified, *Cyperus* sequence (DQ006178.1). The ITS sequence obtained from *nnjem lwami* had *Panicum dichotomiflorum* Michx. as its best match, but with only 94% sequence similarity. The ITS sequence was 90% identical to an ITS sequence from *E. repens*, and 84% identical to our *C. dactylon* ITS reference sequence.

Oudn lhlouff
Oudn lhlouff is not mentioned by Bellakhdar [1]. The market sample we tested matched with 100% similarity for rpoC1 and ITS with sequences from a vouchered reference of *Pulicaria odora* (L.) Rchb. *P. odora* is common in Morocco [4].

Sargina
S*argina* is a Berber vernacular name that refers to *Corrigiola litoralis* subsp. *telephiifolia* (Pourr.) Briq. [1]. This subspecies of *C. litoralis* that can be distinguished from the mostly annual *C. litoralis* subsp. *litoralis* on the basis of its thick woody roots [23]. The Arab name, *bahur al-barbar*, refers to the use of the plant by the Berber people as a perfume [1]. Six market samples of *sargina* were analyzed: one traded as *sargina*, two as normal *sargina* (*l3adia*), two as hollow *sargina* (*lmsouswa*), and one as *sargina rrhamania*, i.e. from Skhour Rehamna. The two samples of *sargina lmsouswa* and one sample of *sargina l3adia* could be identified as *C. litoralis* subsp. *telephiifolia* on the basis respectively all three markers, rpoC1, and psbA. The sample that was sold as plain *sargina*, was identified as *C. litoralis* subsp. *litoralis*. The other sample of *sargina l3adia* is probably a species of *Silene*, as its ITS sequence was 99% identical to that of *Silene mentagensis* Cosson, a species endemic

to Morocco. The *sargina rrhamania* sample could be identified as Caryophyllaceae, but it is not clear to which genus it belongs. Both subspecies of *C. litoralis* are common in Morocco [5].

Ssder
Ssder, the Arab name used for *Ziziphus lotus* (L.) Lam., is a product used to treat fever, according to Bellakhdar [1]. This is confirmed on the basis of our rpoC1 and psbA sequence data. The sequences obtained from the market sample were identical to the sequences from the *Z. lotus* reference specimen, and more dissimilar from sequences obtained from closely related *Ziziphus* sequences. *Z. lotus* is not threatened in Morocco [2].

Tafga
Tafga is the Arabic name for *Centaurea chamaerhaponticum* Ball (= *Rhaponticum acaule* (L.) DC) [1]. Two samples of *tafga* were analyzed, and one could be identified as *R. acaule* on the basis of its ITS sequence, but the psbA sequence was only 94% identical to the *R. acaule* reference sequence. The rpoC1 sequence was highly similar to sequences from several genera of Asteraceae. The second sample yielded only rpoC1 and psbA sequences, and it could only be identified to the family level as Asteraceae. *R. acaule* is common everywhere in the Maghreb [2–6].

Talh
Talh refers to several species of *Acacia*, but is mainly used for *A. raddiana* Savi (= *A. tortilis* subsp. *raddiana* (Savi) Brenan) [1]. Three samples of *talh* were analyzed; one generic, one male (*dkr*), and one female (*ntwa*). *Acacia* species have hermaphroditic flowers, and the reference to the sex of the plants is hence probably not related to the sex of the plant. All samples yielded rpoC1 and psbA sequences that are identical to those of *A. gummifera* L., and somewhat different from sequences that were obtained from other species of *Acacia*. Bellakdhar [1] reports that *A. gummifera* is used in the same way as *A. tortilis* subsp. *raddiana*. *A. gummifera* is endemic to Morocco [6].

Taskra
Taskra could be confirmed to be a species of *Echinops*, which is in agreement with Bellakhdar [1], that lists it as *E. spinosus* L. (= *E. spinosissimus* Turra), but a species level identification on the basis of the sequence data is not possible. Two species of *Echinops*, *E. spinossissimus* and *E. strigosus* L., occur in Morocco, and neither of them is rare [2].

Terta
According to Bellakhdar [1], *terta* is used exclusively for *Withania frutescens* (L.) Pauquy. Out of the three analyzed samples of *terta*, two were certainly a species of *Withania*. The third could be identified as *Kundmannia sicula* (L.) DC., but this is usually traded as *zziyata* [1]. None of the species of *Withania* or *Kundmannia* occurring Morocco are rare or threatened [2].

Tigigest

Tigigest refers to several species in Caryophyllaceae genera *Silene* and *Saponaria* [1]. The analyzed market sample could be identified as *Silene vulgaris* (Moench) Garcke. The Flore pratique du Maroc [5] lists 71 species of *Silene* and one species of *Saponaria* for Morocco. *S. vulgaris* is common almost everywhere in Europe and Northern Africa [2].

Zziyata

Zziyata is a name that can refer to several different plant products, *Kundmannia sicula* (L.) DC., *Limoniastrum guyonianum* Durieu ex Boiss., *L. ifniense* (Caball.) Font Quer, *Conium maculatum* L. *Helosciadium nodiflorum* (L.) Koch. (= *Apium nodiflorum* (L.) Lag.), and *Polygonum maritimum* L. [1]. The single market sample could be identified as *K. sicula* on the basis of its rpoC1 and ITS sequences. Analysis of additional market accessions of *zziyata* could provide insight into whether these other species are commercialized under this name.

References Supplemental data S1

1. Bellakhdar J (1997) La pharmacopée marocaine traditionnelle: Médecine arabe ancienne et savoirs populaires. Saint-Etienne, France: Ibis. 764 p.
2. Fennane M, Ibn Tattou M (1998) Catalogue des plantes vasculaires rares, menacées ou endémiques du Maroc. Bocconea 8: 1–243.
3. Fennane M, Ibn Tattou M (2005) Flore vasculaire du Maroc: Inventaire et chorologie 1. Rabat, Morocco: Institut Scientifique, Université Mohammed V. 483 p.
4. Ibn Tattou M, Fennane M (2008) Flore vasculaire du Maroc: Inventarie et chorologie 2. Rabat, Morocco: Institut Scientifique, Université Mohammed V. 398 p.
5. Fennane M, Ibn Tattou M, Mathez J, Ouyahya A, El Oualidi J, editors (1999) Flore pratique du Maroc. Volume 1. Rabat, Morocco: Institut Scientifique, Université Mohammed V. 558 p.
6. Fennane M, Ibn Tattou M, Ouyahya A, El Oualidi J, editors (2007) Flore pratique du Maroc. Volume 2. Rabat, Morocco: Institut Scientifique, Université Mohammed V. 636 p.
7. Pujadas-Salvà AJ, Plaza-Arregui L (2003) Studies on Thapsia (Apiaceae) from north-western Africa: a forgotten and a new species. Bot J Linn Soc 143: 433–442.
8. Smitt UW, Jäger AK, Adsersen A, Gudiksen L (1995) Comparative studies in phytochemistry and fruit anatomy of Thapsia garganica and T. transtagana, Apiaceae (Umbelliferae). Bot J Linn Soc 117: 281–292.
9. Daniele C, Dahamna S, Firuzi O, Sekfali N, Saso L, et al. (2005) Atractylis gummifera L. poisoning: an ethnopharmacological review. J Ethnopharm 97: 175–181. doi:10.1016/j.jep.2004.11.025.
10. Roger B, Jeannot V, Fernandez X, Cerantola S, Chahboun J (2011) Characterisation and Quantification of Flavonoids in Iris germanica L. and Iris pallida Lam. Resinoids from Morocco. Phytochem Analysis: Online First. doi:10.1002/pca.1379.
11. Tutin TG, Heywood VH, Burges NA, Moore DM, Valentine DH, et al. (1972) Flora Europaea: Volumes 1-5. Cambridge, UK.: Cambridge University Press. p.

12. Tu T, Volis S, Dillon MO, Sun H, Wen J (2010) Dispersals of Hyoscyameae and Mandragoreae (Solanaceae) from the New World to Eurasia in the early Miocene and their biogeographic diversification within Eurasia. Mol Phylogenet Evol 57: 1226–1237. doi:10.1016/j.ympev.2010.09.007.
13. El Beyrouthy M, Marc EB, Arnold N, Nelly A, Delelis-Dusollier A, et al. (2008) Plants used as remedies antirheumatic and antineuralgic in the traditional medicine of Lebanon. J Ethnopharm 120: 315–334. doi:10.1016/j.jep.2008.08.024.
14. Boumerfeg S, Baghiani A, Messaoudi D, Khennouf S, Arrar L (2009) Antioxidant properties and xanthine oxidase inhibitory effects of Tamus communis L . root extracts. Phytother Res 23: 283–288. doi:10.1002/ptr.
15. Paoletti MG, Dreon A, Lorenzoni G (1995) Pistic, traditional food from western Friuli, NE Italy. Econ Bot 49: 26–30.
16. Bellakhdar J, Honda G, Miki W (1982) Herb drugs and herbalists in the Maghrib. Tokyo, Japan: Institute for the Study of Languages and Cultures of Asia and Africa. 339 p.
17. Boulos L (1983) Medicinal plants of North Africa. Algonac, Michigan: Reference Publications Inc. 286 p.
18. Denk T, Grimm GW (2010) The oaks of western Eurasia: Traditional classifications and evidence from two nuclear markers. Taxon 59: 351–366.
19. Duke JA (2011) Dr. Duke's Phytochemical and Ethnobotanical Database, Phytochemical Database. Available:http://www.ars-grin.gov/duke/. Accessed 1 February 2011.
20. Frison G, Favretto D, Zancanaro F, Fazzin G, Ferrara SD (2008) A case of beta-carboline alkaloid intoxication following ingestion of Peganum harmala seed extract. Forensic Sci Int 179: e37–43. doi:10.1016/j.forsciint.2008.05.003.
21. Calviño CI, Martínez SG, Downie SR (2008) The evolutionary history of Eryngium (Apiaceae, Saniculoideae): rapid radiations, long distance dispersals, and hybridizations. Mol Phylogenet Evol 46: 1129–1150. doi:10.1016/j.ympev.2007.10.021.
22. Calviño CI, Martínez SG, Downie SR (2008) Morphology and biogeography of Apiaceae subfamily Saniculoideae as inferred by phylogenetic analysis of molecular data. Am J Bot 95: 196–214. doi:10.3732/ajb.95.2.196.
23. Tutin TG, Burges NA, Chater AO, Heywood VH, Moore DM, et al. (1993) Flora Europaea, 2nd Edition, Volumes 1-5. Cambridge, UK.: Cambridge University Press. p.
